# Potential ecotoxicological effects of antimicrobial surface coatings: a literature survey backed up by analysis of market reports

**DOI:** 10.7717/peerj.6315

**Published:** 2019-02-11

**Authors:** Merilin Rosenberg, Krunoslav Ilić, Katre Juganson, Angela Ivask, Merja Ahonen, Ivana Vinković Vrček, Anne Kahru

**Affiliations:** 1 Laboratory of Environmental Toxicology, National Institute of Chemical Physics and Biophysics, Tallinn, Estonia; 2 Department of Chemistry and Biotechnology, Tallinn University of Technology, Tallinn, Estonia; 3 Institute for Medical Research and Occupational Health, Zagreb, Croatia; 4 Faculty of Technology, Satakunta University of Applied Sciences, Rauma, Finland; 5 Estonian Academy of Sciences, Tallinn, Estonia

**Keywords:** Antimicrobial coatings, Antifouling coatings, Environmental effects, Release, Literature analysis, Safe-by-design

## Abstract

This review was initiated by the COST action CA15114 AMICI “Anti-Microbial Coating Innovations to prevent infectious diseases,” where one important aspect is to analyze ecotoxicological impacts of antimicrobial coatings (AMCs) to ensure their sustainable use. Scopus database was used to collect scientific literature on the types and uses of AMCs, while market reports were used to collect data on production volumes. Special attention was paid on data obtained for the release of the most prevalent ingredients of AMCs into the aqueous phase that was used as the proxy for their possible ecotoxicological effects. Based on the critical analysis of 2,720 papers, it can be concluded that silver-based AMCs are by far the most studied and used coatings followed by those based on titanium, copper, zinc, chitosan and quaternary ammonium compounds. The literature analysis pointed to biomedicine, followed by marine industry, construction industry (paints), food industry and textiles as the main fields of application of AMCs. The published data on ecotoxicological effects of AMCs was scarce, and also only a small number of the papers provided information on release of antimicrobial ingredients from AMCs. The available release data allowed to conclude that silver, copper and zinc are often released in substantial amounts (up to 100%) from the coatings to the aqueous environment. Chitosan and titanium were mostly not used as active released ingredients in AMCs, but rather as carriers for other release-based antimicrobial ingredients (e.g., conventional antibiotics). While minimizing the prevalence of healthcare-associated infections appeared to be the most prosperous field of AMCs application, the release of environmentally hazardous ingredients of AMCs into hospital wastewaters and thus, also the environmental risks associated with AMCs, comprise currently only a fraction of the release and risks of traditional disinfectants. However, being proactive, while the use of antimicrobial/antifouling coatings could currently pose ecotoxicological effects mainly in marine applications, the broad use of AMCs in other applications like medicine, food packaging and textiles should be postponed until reaching evidences on the (i) profound efficiency of these materials in controlling the spread of pathogenic microbes and (ii) safety of AMCs for the human and ecosystems.

## Introduction

The undesirable growth of (micro)organisms on solid surfaces, that is, biofouling or biocontamination of surfaces represents an important threat in diverse surface settings, for example, medical implants (Hetrick & Schoenfisch, 2006), water purification (Nguyen, Roddick & Fan, 2012) or food storage (Hannon et al., 2017). The first successful attempts to control the biofouling by using biocidal surface coatings (i.e., surfaces amended with chemical substances that can destroy or control the undesirable organisms)—mostly by slow controlled leaching of copper from copper compounds—date back to several centuries and were used in navy for painting the ship hulls to control the growth of algae and barnacles and subsequently to enhance the boat’s technical properties and reduce fuel consumption (Yebra, Kiil & Dam-Johansen, 2004; Martins et al., 2018). In early 1960s, tributyltin-based ship paints were successfully introduced, but were phased out and then prohibited in 2008 due to their ecotoxicological effects (Dafforn, Lewis & Johnston, 2011).

Nowadays, Cu(I)-based biocidal pigments either alone or together with ZnO or organic algaecides are main biocidal agents applied to ships (Turner, 2010; Martins et al., 2018). However, antifouling surfaces are not only used on ships but in a great variety of other applications; if these surfaces are meant to control the spread of microbes, these are defined as antimicrobial surfaces.

The development, application and use of AMCs is nowadays a widespread and fast-growing area, powered by nanotechnology. The international antimicrobial council has forecasted that the global market of AMCs will reach $4.5 billion and nearly 590 kilotons of production volume by 2020 (International Antimicrobial Council, 2015).

Historically, the first generation of the AMCs was “release-based” (analogously to antifouling ship hull paints) and was mainly applied on surfaces that were impregnated either with antibiotics or silver-containing compounds (Cloutier, Mantovani & Rosei, 2015). Nowadays, there are many different chemical strategies and technologies to obtain AMCs including application of agents that become active (i) after eluting metallic ions or nanoparticles (NPs), antibiotics, chloride, iodine (i.e., are release-based), or (ii) upon contact with surface-anchored compounds (e.g., cationic compounds such as quaternary ammonium compounds (QACs) and chitosan), or (iii) upon exposure to UV-light like TiO_2_ (Grass, Rensing & Solioz, 2011; Dizaj et al., 2014; Chen et al., 2014). Additional strategy is to produce “anti-adhesion” surfaces that prevent biofilms formation due to the physical properties of surfaces (for thorough review and references therein, see Cloutier, Mantovani & Rosei, 2015). Thus, as a large group of AMCs is based on surface-releasing compounds, and aquatic phase represents one of the main pathways how toxicants are “travelling” through the environment, entrance of biocidal compounds from AMCs to aquatic environment should be carefully considered as a part of the risk assessment process. After reaching water bodies, AMCs ingredients may easily further spread to other environmental compartments (water, soil, sediments), where they could pose a threat to different organisms inhabiting these compartments. However, this issue has been addressed only in a few papers aiming to find more environmental-friendly approaches to replace tributyltin-based ship paints (Selim et al., 2017). Today, there are no general and mutual prospects about the ecotoxicological threats of AMCs used in various applications.

One of the tasks of the COST Action network AMICI is to weigh the pros and cons of AMCs by analyzing the antimicrobial efficiency by means of healthcare-associated infections (HAIs) reduction vs. outweighing the negative consequences that may occur to the natural environment/ecosystems during various life cycle stages of AMCs and respective ingredients (production, transportation, use and waste-depositing). Being a part of this COST Action network, we carried out a literature survey where we focused on AMCs containing silver, titanium, copper, zinc, chitosan and QACs and paid special attention to the release of the active ingredients into the aqueous phase as a proxy of their potential to induce ecotoxicological effects. Keeping in mind the diverse applicability of universal properties of biocides the literature survey was not limited by intended application of the AMCs as the same ingredients released from different applications are expected to have ecotoxicological impact.

### Methodology of the literature survey

**Figure 1:**
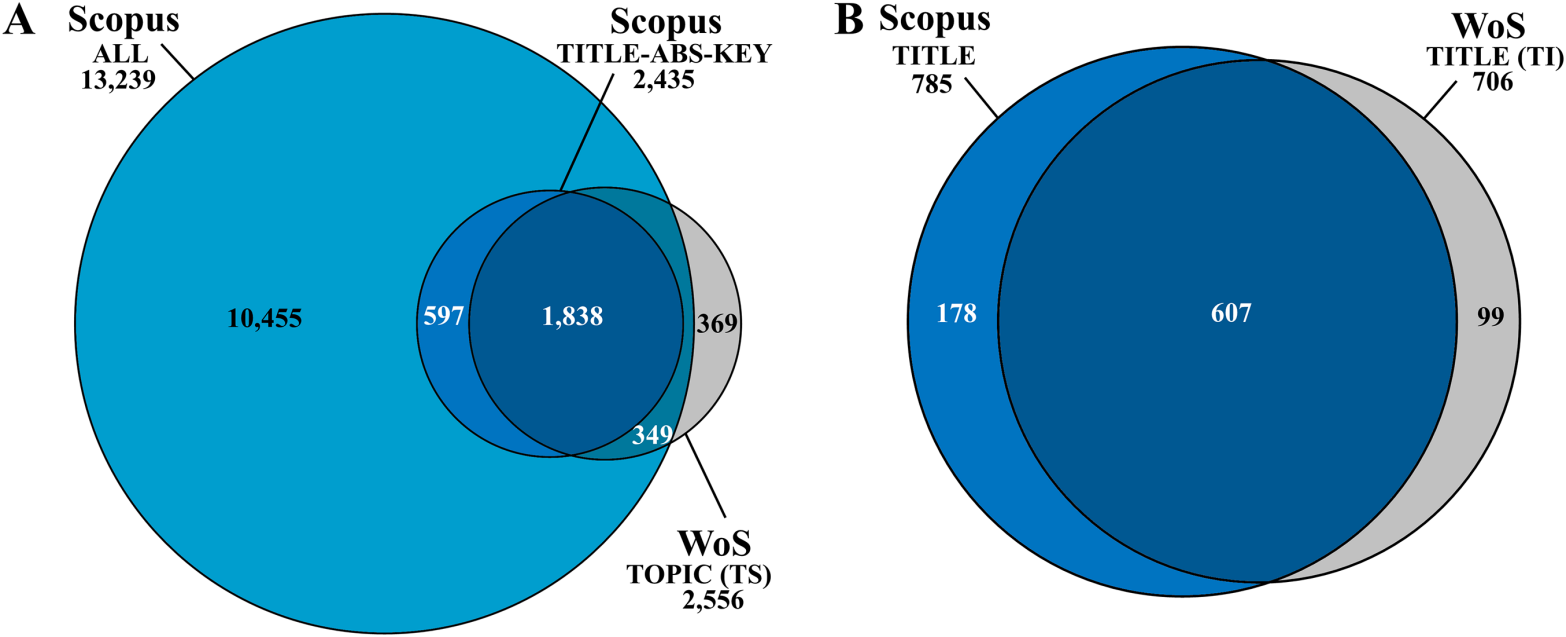
Venn diagrams of coverage and overlapping of articles about antimicrobial/antifouling surface coatings collected in Scopus and Web of Science (WoS) databases. (A) Comparison between results obtained with search fields Scopus “all,” Scopus “title-abstract-keywords,” and WoS “topic.” (B) Comparison between results obtained with search fields Scopus “title” and WoS “title.” The searches were performed on May 28 and May 31, 2018 using search phrases listed in Table S1 (given in File S1).

Literature survey was performed between April and July 2018 in Scopus, as the coverage of Scopus on the number of papers in most disciplines is a bit higher than in another widely used Web of Science (WoS) database (Harzing & Alakangas, 2016). In addition, Scopus covers larger proportion of journals in the fields of Biomedical Research and Natural Sciences and Engineering than WoS database (Mongeon & Paul-Hus, 2016). The Google Scholar database was omitted from our survey although its coverage is even higher, but there might be many duplicate records and the quality of indexed material is more variable as the quality control process is not as strong as in Scopus or WoS (Mongeon & Paul-Hus, 2016; Harzing & Alakangas, 2016). A literature search performed for the current study using search phrases listed in the Table S1 revealed that the coverage of papers about antimicrobial/antifouling coatings is similar in both Scopus and WoS (Fig. 1). While both of these databases enable searches in the fields of title, abstract and keywords, they add index keywords generated on different bases. Differently from WoS, a search from all fields could be performed in Scopus (Fig. 1A). The only search field which exists both in WoS and Scopus and thus enables comparison of these two databases is the field “title.” Comparison of the results obtained with phrase search in the field of “title” suggested that Scopus incorporates larger number of relevant papers (Fig. 1B). Therefore, search in Scopus was selected for further analysis. The scientific quality of retrieved papers was limited by Scopus database indexing criteria so that only peer-reviewed literature was used in the survey.

**Figure 2:**
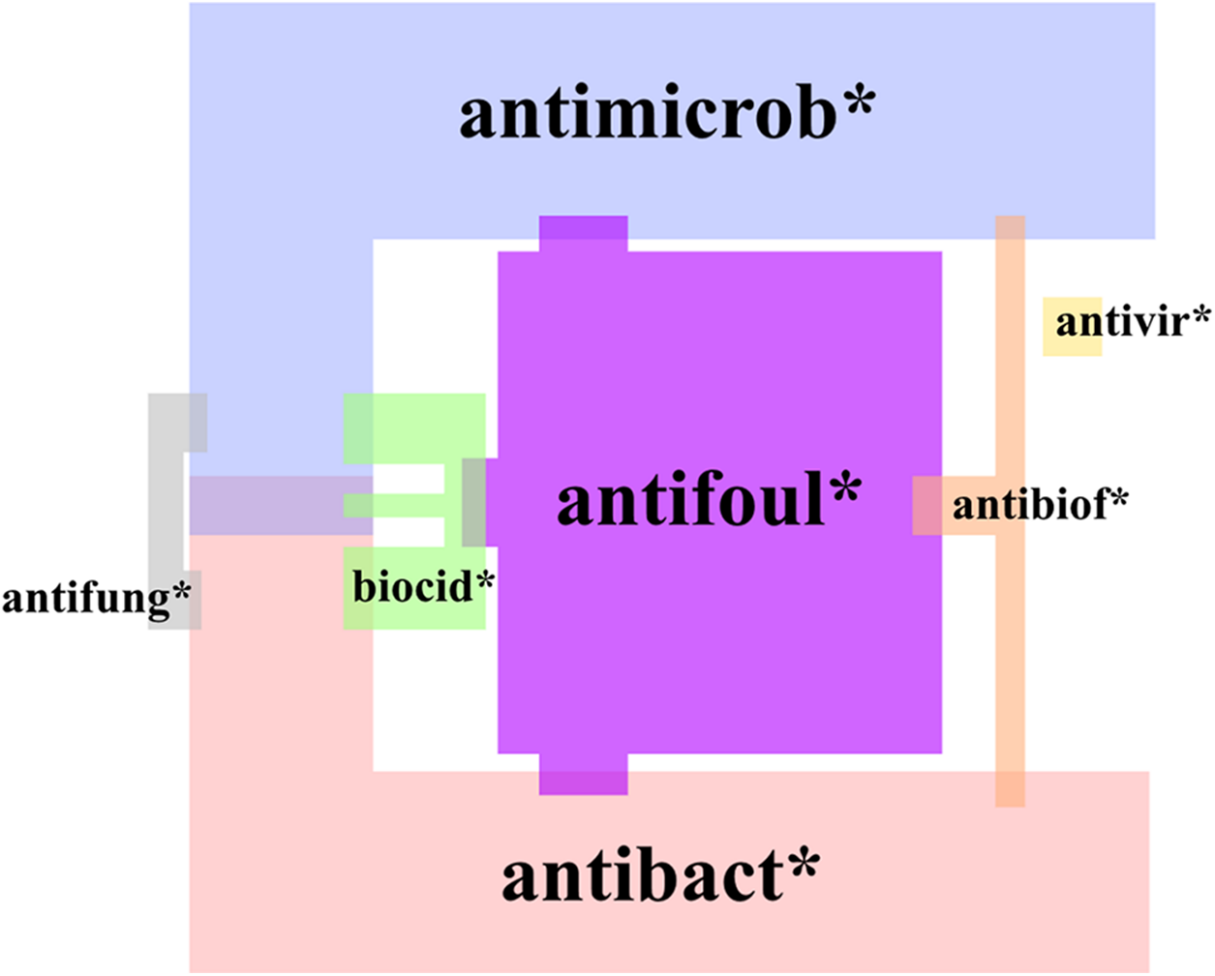
Proportions and overlap of articles on antimicrobial surface coatings found using different (truncated) search phrases. The size of the respective color block surface is proportional to the number of papers. Total number of papers was 2,427. Search was performed in Scopus on May 4, 2018.

A crucial step when performing a literature survey is the selection of search phrases. To ensure that all ingredients used in AMCs were given equal weight, no material-specific terms were used in the initial search, but only search terms referring to antimicrobial nature and coating were included. In order to involve the most relevant papers, we considered the following terms: antimicrobial (antimicrob*), antibacterial (antibact*), biocidal (biocid*), antiviral (antivir*), antifungal (antifung*), anti(bio)fouling (antibiof*, antifoul*), coatings (coat*) and surfaces (surface*). Truncated search terms were used as these enabled us to find relevant papers with alternate endings of the respective terms. All terms were searched as two-word phrases by applying quotation marks to avoid irrelevant papers (e.g., about surface coatings of nanomaterials, surface water, etc.). Indeed, the analysis of the papers retrieved on May 4, 2018 applying this strategy indicated that the chosen terms were rarely used as synonyms as overlap between them was rather marginal (Fig. 2). The largest body of papers was obtained with search phrases containing antimicrob*, antifoul* and antibact*; whereas the phrase “antivir* coat*” OR “antivir* surface*” gave only 10 results. The latter papers were also the only papers that did not overlap with any papers obtained with other search terms. Highest overlap was observed among papers found with search phrases containing antimicrob* and antibact* (Fig. 2).

In order to quantify the prevalence of various chemical ingredients used in AMCs, their main biological targets, and areas of application, word and phrase (two to five words) frequency was analyzed by using the titles, abstracts and keywords of the papers retrieved from the Scopus. Resulting words and phrases with occurrence of more than 10 times were thematically categorized into three groups: material, target and application (see File S2). For further analysis, the redundant phrases were merged, and the prevalence of remaining relevant words and phrases was illustrated with word clouds (generated in https://www.wordclouds.com/). All search term combinations used for literature survey methodology are given in Supplemental Files.

### Antimicrobial surface coatings: materials, targets and applications

For environmental risk assessment of AMCs, both the hazard of AMC ingredients (i.e., the concentration range of the compound that is toxic to certain environmental organism or a group of organisms) and their environmental levels should be assessed and considered. As a rule, the ecotoxicity evaluation of a chemical compound relies on tests conducted with model organisms that belong to aquatic ecosystem. Usually, organisms from different aquatic food web levels are used: algae, crustaceans (mostly daphnids), and fish. The most sensitive organism (i.e., weakest link in the ecosystem) will “rank” the compound according to its toxicity (harmful, toxic, very toxic, etc.).

As an example, the concentration ranges of the classification/aquatic toxicity ranking of industrial chemicals according to EU-Directive 93/67/EEC (CEC, 1996) were based on the lowest median L(E)C50 value of the three key environmental organisms: algae, crustaceans and fish. <1 mg/L = very toxic to aquatic organisms; 1–10 mg/L = toxic to aquatic organisms; 10–100 mg/L = harmful to aquatic organisms; >100 mg/L = not classified.

In spite of existing methods for hazard assessment of individual chemical compounds, determination of the amounts in which these chemicals are used and get into contact with the organisms from different environmental compartments (water, soil, sediments) still poses a challenge. The latter depends also on the field of application and the product life cycle—while some AMCs, for example, those used in the marine industry for ship paints are in direct contact with the surrounding aquatic environment during their intended use, others, such as AMCs used in healthcare settings, could encounter the surface water, soil or sediment only during production phase, after disposal or via active substance (i.e., the substance responsible for antimicrobial effect) release during, for example, the cleaning process.

To identify the main ingredients of AMCs and their major applications, two complementary approaches were used: (i) meta-analysis of the published scientific literature, which reflects scientific interest and potential future trends of AMCs and (ii) overview of the data on production volumes of AMCs that can be obtained from market reports but are unfortunately rare and of limited access.

#### Major chemical ingredients, microbial targets and applications of AMCs

**Figure 3:**
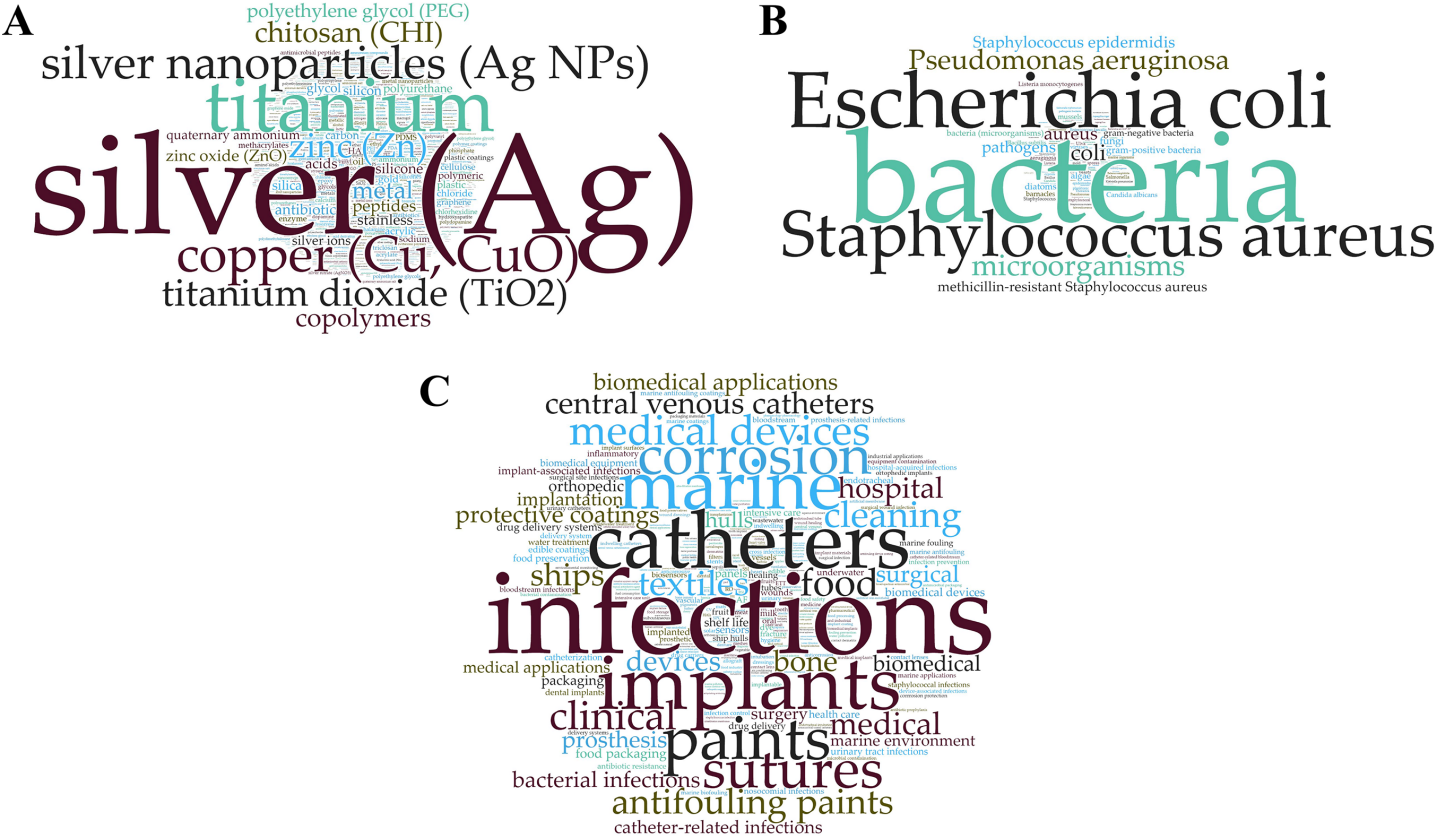
Major chemical ingredients (A), target organisms (B) and application areas (C) of antimicrobial surface coatings. Panels A–C (word clouds) are based on the bibliometric study conducted in Scopus on April 4, 2018 (File S2).

The major chemical ingredients (A), biological targets (B) and application areas (C) of AMCs are depicted as word clouds in the Fig. 3, where the size of the word reflects its abundance in the titles, abstracts and keywords of the papers retrieved with the search performed in Scopus on April 4, 2018 (search phrase in Table S1 “Scopus, TITLE-ABS-KEY” of File S1). Altogether 2,410 papers were analyzed to obtain these word clouds. Word and phrase frequency analysis showed that the majority of the AMCs contained silver as the antimicrobial agent (Fig. 3A) and the main targets of AMCs were bacteria (Fig. 3B) although silver-based NPs have also been shown to be antiviral (Park et al., 2014). AMCs against viruses and fungi were significantly less frequent, most likely due to the complex nature of these studies. This is also further confirmed by the fact that there is no overlapping between antiviral and antibacterial/antimicrobial surfaces indicating separate field of research and methodology that is not reported together in the same papers. However, results on antiviral efficacy of known antibacterial materials, for example, copper containing AMCs against norovirus and influenza A have been published (Warnes, Summersgill & Keevil, 2015; Noyce, Michels & Keevil, 2007). While the major intended applications of AMCs seemed to be healthcare related, that is, infections, catheters, implants, these were tightly followed by other areas like applications in marine settings, paints, food and textiles (Fig. 3C).

**Figure 4:**
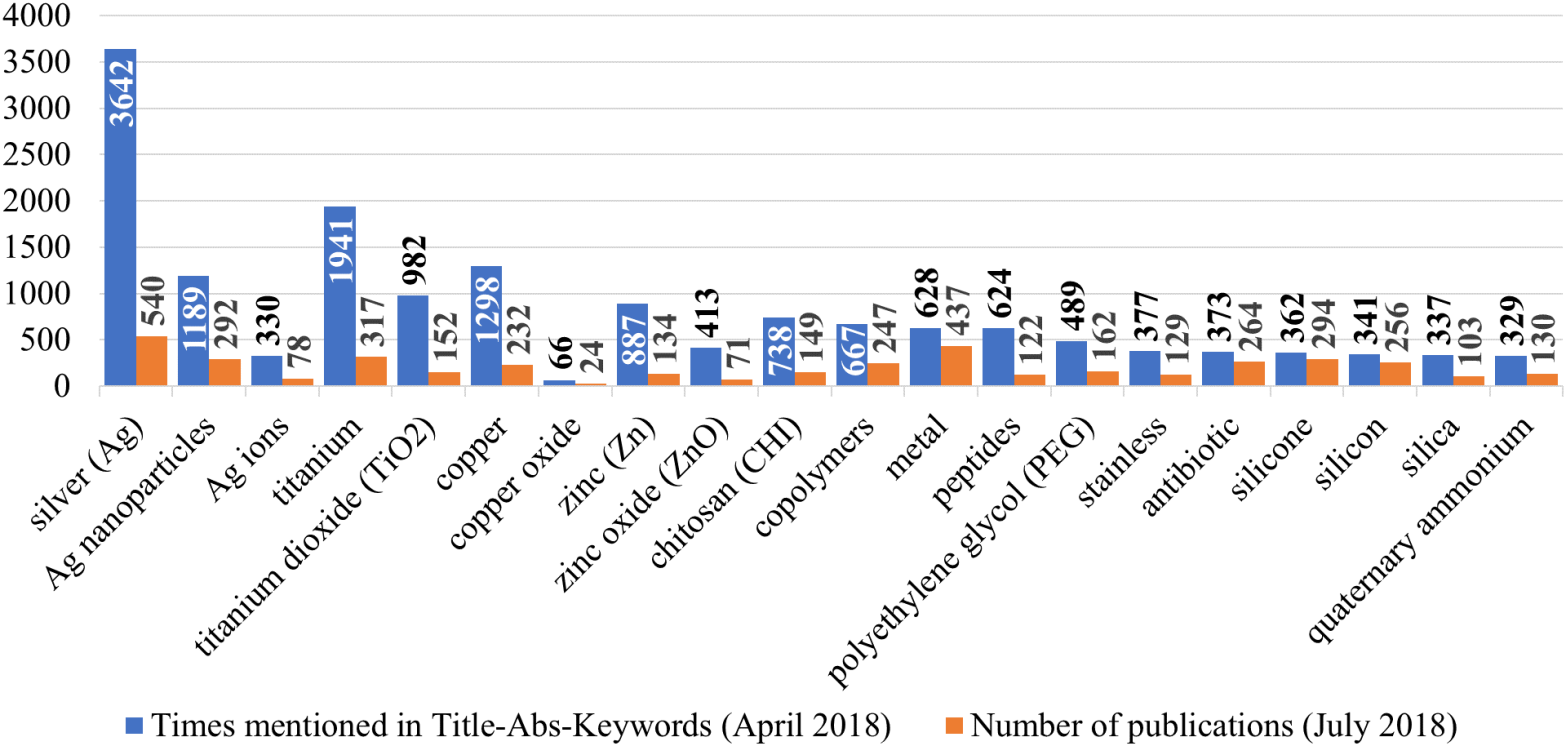
The most prevalent ingredients of antimicrobial surface coatings. Prevalence according to the number of times mentioned in title, abstract and keywords (blue columns) of the papers retrieved on April 4, 2018 (search phrase in Table S1 “Scopus, TITLE-ABS-KEY” of File S1). The number of publications (orange columns) in Scopus for respective materials on July 21, 2018 (search phrases in Table S2 of File S1, total number of publications was 2,720).

The search with the same search phrase (see Table S1 “Scopus, TITLE-ABS-KEY” and Table S2 “total” of File S1) performed again on July 21, 2018 resulted in 2,720 papers, out of which 20% (540 papers) were focused on Ag-based AMCs. More than a half (54%) of these papers described Ag NP-based coatings, while only 14% of them mentioned Ag ions or Ag salts (Fig. 4). The high prevalence of Ag in AMCs was expected as Ag has been used as a biocidal agent for centuries (Nowack, Krug & Height, 2011). Another material with the high prevalence in AMCs was titanium (317 papers; 12%), probably due to the fact that titanium and its alloys are common implant materials (Saini, 2015). Metals were generally often used in AMCs: 437 papers (16%) mentioned “metal” in its title, abstract or keywords. Besides Ag and Ti, other metals commonly used in AMCs were copper (232 papers; 9%) and zinc (134 papers; 5%) (Fig. 4). Both metals have long history in various antimicrobial applications. They are widely used in commercial marine antifouling paints and coatings, especially after the restriction of use of tributyltin and other organotin compounds, which were banned internationally by 2008 due to their harmful effects to the marine organisms (IMO, 2001). Copper re-emerged in marine antifouling applications (Gipperth, 2009), and is often used in combination with zinc in self-polishing antifouling coatings (Yebra, Kiil & Dam-Johansen, 2004). Interestingly, while 53% of zinc-related papers were about zinc oxide, only 10% of copper-related papers investigated copper oxides (Fig. 4).

Application of silicone (294 papers; 11%), silicon (256 papers; 9%), and silica (103 papers; 4%) (Fig. 4) in AMCs was also quite frequent, but mainly as a substrate (Fu et al., 2018; Grumezescu et al., 2018) or an additive to control the release of other antimicrobial substances (Cazacu et al., 2012; Li et al., 2013; Ruggiero et al., 2018).The number of papers on traditional antibiotics (i.e., antimicrobial agents targeted specifically against bacteria and typically used in human and veterinary medicine to fight bacterial infections) usage in AMCs was rather high: 10% (264) of all retrieved papers (Fig. 4). On the other hand, none of these were individually comparable with the major materials—vancomycin was mentioned in 44 papers (1.6%) gentamicin in 40 papers (1.5%), while rifampicin, minocycline, ciprofloxacin, sulfadiazine, penicillin, tetracycline and ampicillin were mentioned in 10–27 papers (less than 1%). Copolymers were used in 9% of the papers (247), but this term reflected a wide variety of polymers that were used together and had various purposes ranging from matrix components and additional layers to reduce dissolution in complex AMCs to independent antifouling coatings. Similarly, polyethylene glycol (PEG, 6% of the papers; 162) was also used as a matrix (Ashraf & Edwin, 2016) or antifouling layer (Lee, In & Park, 2014; He et al., 2016b) in AMCs rather than active biocidal ingredient (Fig. 4).

Both chitosan and QACs were mentioned in ca. 5% of the retrieved papers (149 and 130 papers, respectively) (Fig. 4). Chitosan is known as biocompatible and biodegradable antimicrobial material with low solubility at physiological pH (Pillai, Paul & Sharma, 2009), but it has also been described as efficient carrier for other release-based biocides (Sinha et al., 2004). QACs are a group of popular antimicrobial substances that exist in a variety of structures (Gerba, 2015). QAC-based coatings have various applications ranging from textiles (Morais, Guedes & Lopes, 2016) to food industry (Galié et al., 2018). “Stainless” referring to stainless steel was also mentioned in 5% of the papers (129), but its major application is as a carrier for AMCs. Finally, (antimicrobial) peptides were studied in 4% of the papers (122) (Fig. 4); however, these are even more diverse group than antibiotics. The most prevalent antimicrobial peptides were melamine (36 papers) and nisin (25 papers).

Surprisingly, triclosan, which is known as an effective agent against a broad spectrum of bacteria due to the inhibition activity of bacterial fatty acid synthesis (McMurry, Oethinger & Levy, 1998), was not found among the top substances that have perspective in AMCs (File S2). This may be explained by no or only limited advantage of triclosan compared to other biocidal agents (Kalyon & Olgun, 2001; Møretrø et al., 2011), or by the fact that microorganisms are prone to develop resistance against triclosan, which resulted also in co-resistance against antibiotics such as tetracycline and erythromycin (Chuanchuen, Narasaki & Schweizer, 2002). Additional concerns regarding triclosan include a wide range of potentially adverse effects, demonstrated *in vitro* and *in vivo*, such as endocrine disruption (Zorrilla et al., 2008; Paul et al., 2009), genotoxicity (Binelli et al., 2009; Lin et al., 2012), immunosuppression (Udoji et al., 2010), and tumor promotion (Yueh et al., 2014; Kim et al., 2015). Moreover, studies have linked allergen sensitization in humans to triclosan exposure (Savage et al., 2012; Bertelsen et al., 2013; Clayton et al., 2010). An additional concern is potential triclosan accumulation in the environment (Heidler & Halden, 2007; Dhillon et al., 2015), particularly in aquatic environment where it may have detrimental impact on animals (Oliveira et al., 2009). Due to these issues, triclosan has received regulatory scrutiny, and its applications have been limited in the EU and the US.

**Figure 5:**
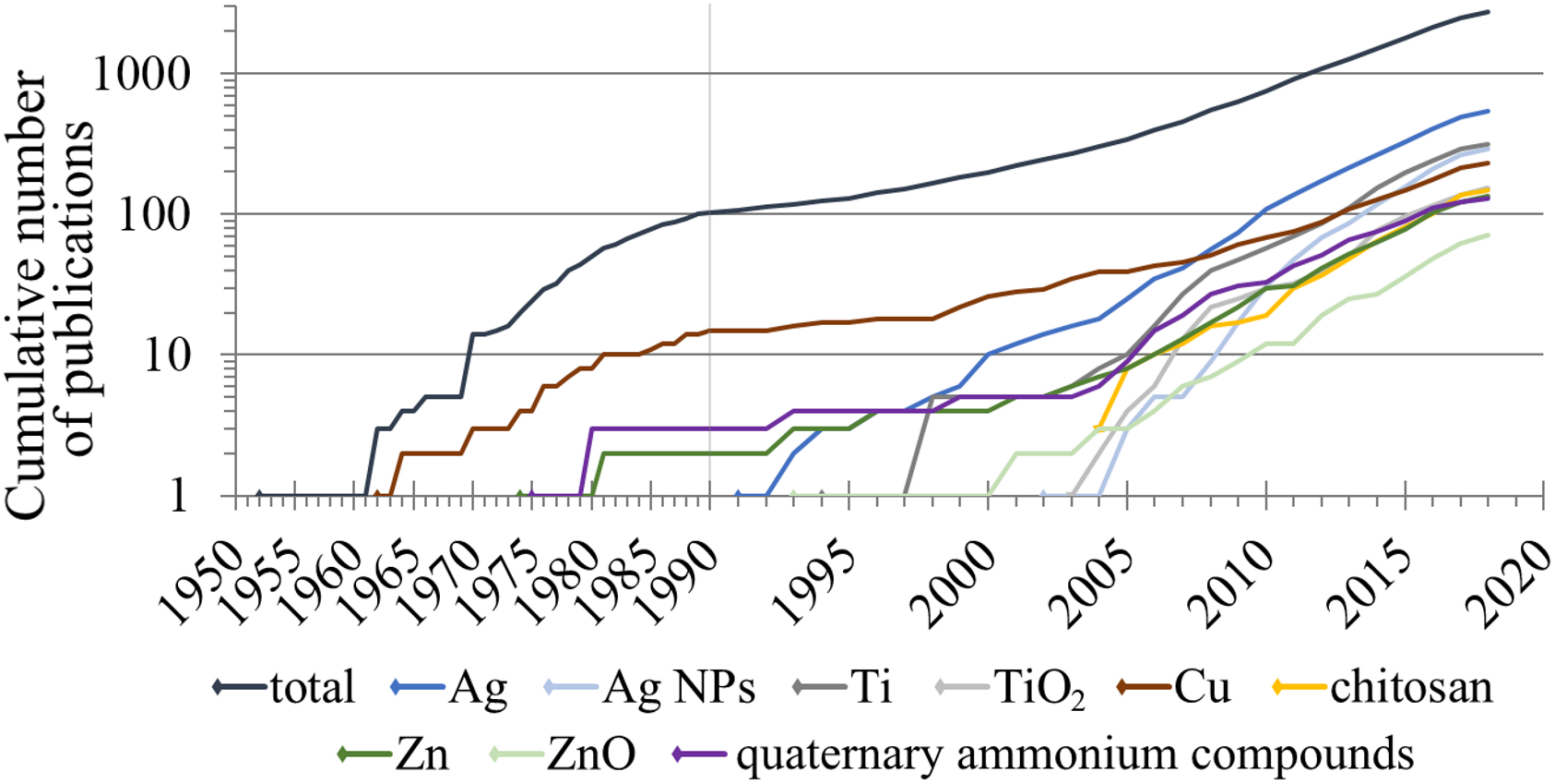
Timeline of evolution of information on different types of antimicrobial surface coatings (in respect of their major ingredients) according to the number of papers in Scopus. The bibliometric data search in Scopus was conducted using the search phrases as indicated in Table S2 on July 21, 2018. Total number of papers (all years): 2,720; see also Fig. 4.

The AMCs-related papers were further analyzed for the appearance of active antimicrobial agents by using additional search terms as indicated in Table S2 (given in File S1). It is interesting to note that frequency and the number of studies on AMCs active ingredients do not reflect the timeline when the use and investigation of these ingredients started (Fig. 5). Namely, the first paper about copper as an antimicrobial agent used in AMCs dated back to 1962 and was followed a decade later by zinc (in 1974) and QACs (in 1975). The first mentioning of silver and titanium in the context of AMCs could be dated back to the beginning of 1990s (Fig. 5) and both metals are currently the major active ingredients of AMCs. Our literature survey showed that the most recently used material for AMCs is chitosan (in 2004), which has gained a lot of interest during the past decade.

**Figure 6:**
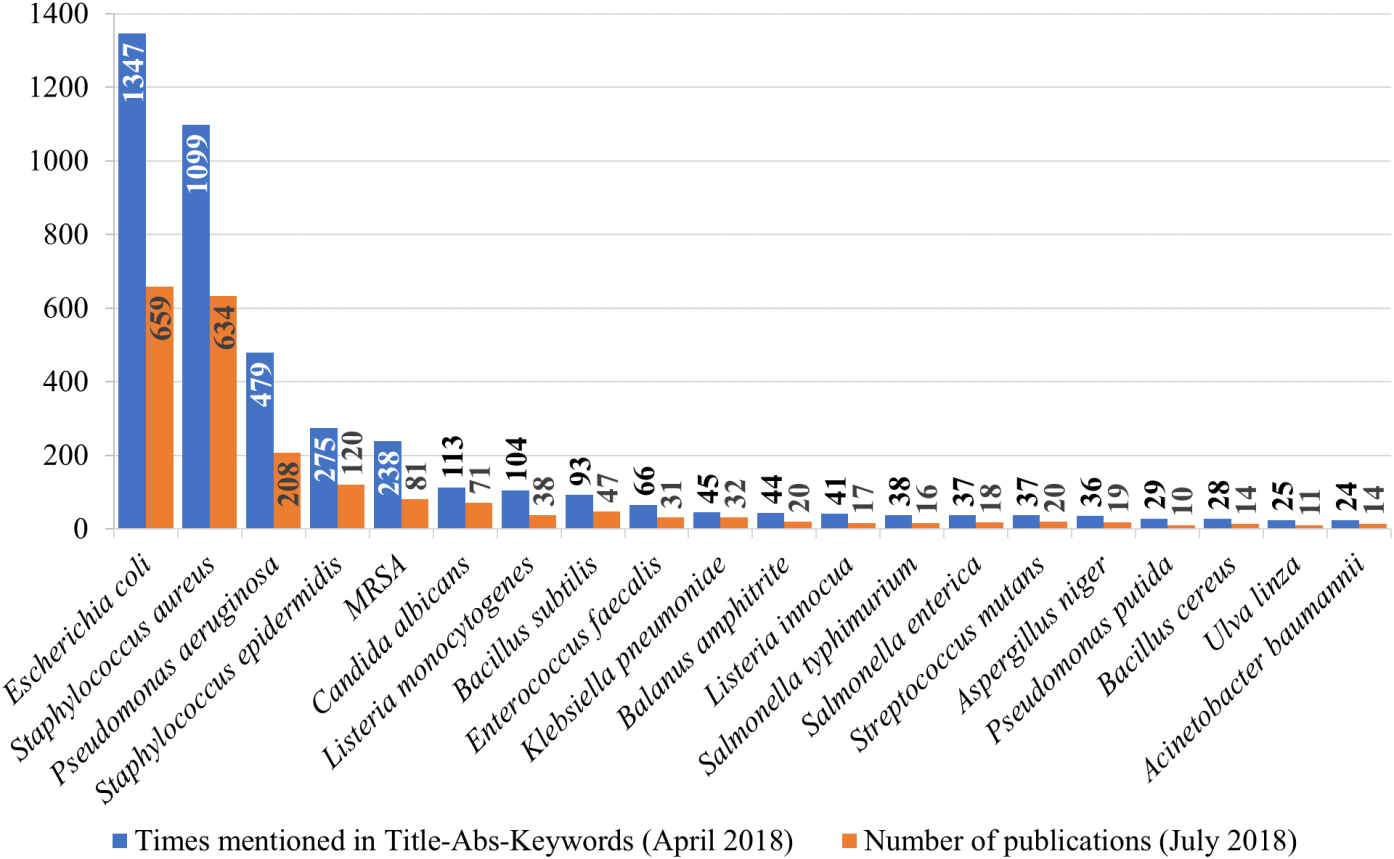
The most prevalent target organisms of antimicrobial surface coatings. MRSA—methicillin-resistant *Staphylococcus aureus*. Prevalence according to the number of times mentioned in title, abstract and keywords (blue columns) of the papers retrieved on April 4, 2018 (search phrase in Table S1 “Scopus, TITLE-ABS-KEY” of File S1). The number of publications (orange columns) in Scopus for respective materials on July 21, 2018 (search phrase Table S2 “total” of File S1 was limited with respective species; total number of publications was 2,720).

As one of the main drivers for AMCs industry is the healthcare sector, challenged by the nosocomial infections or HAIs (Transparency Market Research, 2014), we performed additional bibliometric analysis on AMCs targets. Such infections, caused by pathogens resistant to antibiotics, are acquired in hospitals by around four million people in Europe (Greenhalgh & Walker, 2017) and almost 800,000 in the US annually (Magill et al., 2014), often with fatal outcomes (99,000 annual deaths in the US (Grand View Research, 2013)). The majority of nosocomial infections are caused by only a few bacterial strains: *Staphylococcus aureus*, *Pseudomonas aeruginosa*, *Escherichia coli*, coagulase-negative staphylococci, predominantly *Staphylococcus epidermidis*), *Enterococcus* spp. (predominately *Enterococcus faecalis* and *Enterococcus faecium*) (Greenhalgh & Walker, 2017). Their resistance to both commonly used and last resort antibiotics poses a significant problem in combating HAIs. All these bacteria were also among the most prevalent AMCs targets indicated in the papers retrieved on July 21, 2018 (Fig. 6). The predominant species were *E. coli* (659 papers; 24%), and *Staphylococcus aureus* (634 papers; 23%). In addition, 81 papers (3.0%) studied the effect of AMCs on methicillin-resistant *Staphylococcus aureus. Pseudomonas aeruginosa* (208 papers; 7.6%), and *Staphylococcus epidermidis* (120 papers; 4.4%) were also abundant targets, while substantially less information was available for *Enterococcus faecalis* (31 papers; 1.1%), *Enterococcus faecium* (10 papers; 0.37%). In addition to these, human (opportunistic) pathogens *Klebsiella pneumoniae* (32 papers; 1.2%), *Streptococcus mutans* (20 papers; 0.74%), and *Acinetobacter baumannii* (14 papers; 0.51%), an opportunistic pathogenic yeast *Candida albicans* (71 papers; 2.6%), mold *Aspergillus niger* (19 papers; 0.70%), and foodborne pathogens *Listeria monocytogenes* (38 papers; 1.4%), *Salmonella typhimurium* (16 papers; 0.59%), *Salmonella enterica* (18 papers; 0.66%) and *Bacillus cereus* (14 papers; 0.51%) were often studied as biological targets. Some of the papers also studied the effects on *Bacillus subtilis* (47 papers; 1.7%), *Listeria innocua* (17 papers; 0.63%) and *Pseudomonas putida* (10 papers; 0.37%) models. A small number of papers studied marine organisms—barnacles *Balanus amphitrite* (20 papers, 0.74%) and green algae *Ulva linza* (11 papers, 0.40%) (Fig. 6). Modest representation of marine species could be attributed to a less species-specific approach in marine antifouling coating research as opposed to medical AMCs research with known pathogenic species of interest and standardized protocols using model organisms.

#### AMCs status according to market overviews

Several companies have compiled market overviews about AMCs providing the actual market situation—most prevalent materials, their use, quantities, revenues, etc., and estimated for the future as well. While the information from such overviews is very useful for life cycle analysis of AMCs, they are generally out of reach due to the high costs. Our study used two sample market overviews: “Antimicrobial Coatings Market Analysis and Segment Forecasts to 2020” (Grand View Research, 2013) and “Antimicrobial Coatings Market: Global Forecast, Market Share, Size, Growth and Industry Analysis 2012–2018” (Transparency Market Research, 2014).

Information on AMCs from these market overviews was in general agreement with the data published in scientific papers accessible by Scopus. Namely, AMCs are widely used in diverse applications to prevent growth of bacteria or fungi on surfaces and coatings used in medical settings, indoor air quality, mold remediation, antimicrobial textiles, food packaging and construction. The market overviews revealed that contrary to researchers’ interest (see the bibliometric study of Scopus, Fig. 3C), the largest segment of the AMCs market is indoor air quality with the global production volume reaching 120 kilotons in 2018. However, experts believe that it still holds untapped potential, as environmental regulations in Europe and the US impose stringent standards on expected indoor air quality in, for example, schools and hospitals. The healthcare sector, which appeared to be the most appealing for research (Fig. 3C), accounts for a major portion of AMCs produced annually and is the second largest end-user of AMCs. Presently, nine out of 10 medical devices make use of AMCs, either as a pre-treatment or in finished products (Grand View Research, 2013).

The global AMCs market is estimated to be worth ca. $3 billion and projected to grow to $4.5 billion and 590 kilotons by 2020. However, volatile silver prices and stringent regulation may potentially dampen market growth (Grand View Research, 2013). The four largest AMCs producers, AkzoNobel NV, Sherwin-Williams, Dow Microbial Control and Diamond Vogel, account for almost half of the total AMCs production worldwide, but do not cover all prominent AMCs applications. Many small regional manufacturers, like Arch Lonza, DuPont, AK Coatings Inc., Nippon Paint Co. Ltd. and others that represent also notable share in the AMCs market (Grand View Research, 2013), provide specific products such as coating applications for medical instruments. Geographically, most of the demand for AMCs comes from North America, specifically the US, which accounted for 39% of market volume in 2013. The high consumption is expected to rise due to high standard of living and increasing healthcare expenditure. For the same period, Europe, Pacific Asia and the Rest of the World accounted for 23, 30 and 7.2%, respectively. The strongest future market growth is expected for Pacific Asia, specifically China and India. Although these countries face with increasing HAIs problem similarly to the US and Europe, AMCs use is not yet as widespread as in Western Europe or North America (Grand View Research, 2013).

Commercially available AMCs can be divided into (i) powder coatings, which can be electrostatically layered to various surfaces, and (ii) surface modification coatings that interact with application surface and confer protection against pathogens. In 2012, total production of powder coatings was 131.4 kilotons, while 154.3 kilotons accounted for surface modification coatings. Powder AMCs are routinely utilized in sanitation, food equipment, domestic appliances, medical care, steel furniture industry, etc. (Transparency Market Research, 2014). The most commonly used antimicrobial material is silver, which accounted for 55% of the total powder AMCs in 2012. Other important ingredients in AMCs are copper, bronze, zeolite, silicone and titanium, which are combined with coating substances, such as polymers, and applied to surfaces via spraying, draw down method, thin film coating and spin coating. (Grand View Research, 2013).

### Ecotoxicological effects of antimicrobial surface coatings via release of active antimicrobial compounds into the aquatic environment

The AMCs often contain compounds that either kill the microbes, inhibit their growth or prevent surface colonization. At the same time, most typical active ingredients released from the AMCs such as silver, copper and zinc can be even more toxic to “non-target” environmental species (algae, fish, crustaceans) (Bondarenko et al., 2013) that become exposed to these compounds via various waste-flows during production, use or disposal of AMCs. While the topic of AMCs has gained scientific interest since 1950s and 1960s, and past 20 years have added tens to hundreds relevant scientific papers per year (Fig. 5), the ecotoxicological and/or environmental effects of antimicrobial surfaces are largely unknown. The top environment-related phrases from the titles, abstracts and keywords of the initial pool of retrieved publications (collected on April 4, 2018) are “environmentally friendly” (82 mentions), “environmentally benign” (43 mentions), and “environmental impact” (38 mentions). However, when the context of the respective papers was analyzed in July 2018 by limiting the search phrase Table S2 “total” (given in File S1) with aforementioned phrases (Table S3), it appeared that most of these studies focused on certain area of application. The phrase “environmentally friendly” was present in the abstract of 2.7% (74 papers) and majority of these papers (42 papers) were about marine antifouling coatings. Such trend was likely motivated by the pressure to find environmentally friendly alternatives for tributyltin. Other papers mostly described development of environmentally friendly method for AMCs production or implementation/modification of natural molecules as environmentally friendly source of antimicrobials. Another phrase “environmentally benign” was present in the abstracts of 26 papers (1.0%) with similar trend—half of the papers (13) described development of safer coatings for marine environment, while the rest mainly aimed to find safer conditions for AMCs production. Likewise, phrase “environmental impact” was used in 34 papers out of which 71% (24 papers) discussed the need to control the environmental impact of marine antifouling coatings.

The phrase “low toxicity” appeared in the abstracts of 26 (1%) retrieved papers. Most of them (17 papers) were more focused on the effect in humans/mammals than on environmental health, while two papers aimed to achieve better production processes by using *low toxicity* techniques or precursors. Out of the remaining papers, two papers assessed the toxic effects of active substances from the AMCs like the effects of polymeric 3-alkylpyridinium salts (poly-APS) isolated from the Mediterranean sponge *Reniera sarai* (Faimali et al., 2003), and secondary metabolites of the marine sponge *Haliclona exigua* (Limna Mol, Raveendran & Parameswaran, 2009) against barnacles, and one paper presented the effects of potential antifouling paint components Tween 85, zinc peroxide, chlorhexidine, copper thiocyanate, dichlofluanid and zinc pyrithione against a set of marine organisms (Carteau et al., 2014). One paper raised the question whether AMCs based on TiO_2_ NPs are safe to marine phytoplankton as TiO_2_ NPs themselves were shown to be toxic toward three different marine species (Miller et al., 2012). The phrase “low toxicity” revealed only three papers in which the potential ecotoxic effect of antifouling coatings (with application as ship paints) was actually tested. Namely, sodium benzoate entrapped into silicone inhibited the bacteria from attaching onto surface, likely due to sodium benzoate leaching from the surface coating (Haque, Cutright & Newby, 2005). Yang et al. (2012) found that multilayer polymer coating with low-fouling properties reduced the settling of barnacles *Amphibalanus amphitrite* on glass substrate from 42% to 9.2% and showed that compared to the pristine glass, the dead fraction of barnacle cyprids was lower after being in contact with polymer-functionalized surfaces. Similarly, a study by Wang & Wei (2016) focused on the effect of anti-adhesive polymer coatings on stainless steel substrate and demonstrated that these coatings did not reduce the viability of marine bacteria *Pseudomonas sp.* or barnacles *Amphora coffeaeformis.* While Cu is often involved as a pigment in marine coatings (Turner, 2010), it did not belong to the composition of polymer coatings mentioned above meaning the coatings have low toxic potential (Yang et al., 2012; Wang & Wei, 2016).

In spite of few attempts to ensure the environmental safety of AMCs by introducing polymeric antifouling coatings, very little is known so far on the potential environmental hazard/ecotoxicity of most prevalent AMCs (e.g., Ag-, Cu-, Ti-, chitosan-containing) or those used in areas other than marine coatings. To fill this knowledge gap, we performed a literature search to reveal the magnitude of potential release into the aquatic environment (including dissolution) of active ingredients used in AMCs. For that purpose, the previously used search phrase was modified by adding AND (releas* OR dissol* OR shed*) (exact phrases can be found in File S3) to collect information on release of silver, titanium, zinc, copper, chitosan and QACs between April and June 2018.

The search revealed that the release of antimicrobial active agents from AMCs may affect the shelf-life of such coatings and subsequently lead to environmental contamination. This scenario was already proven for silver leaching from antibacterial textiles during washing cycles (Reed et al., 2016) and indicates the way for contamination of the sewage treatment systems, but also surface waters and other environmental compartments. Mitrano et al. (2016) observed that depending on detergent, up to 75% of silver may be released from textiles impregnated with Ag NPs in one washing cycle, and the Swedish Chemicals Agency reported cases where 98% of silver vanished from antimicrobial textile after >10 washing cycles (Frank, 2012). The US National Institute of Standards and Technology reported about the migration behavior of NPs from flooring finishes and indoor paints after cleaning (Sung, Nguyen & Persily, 2014). Released nano- and microsized particles from surface abrasion of silicon-based nanocoatings and titania-, aluminum-, and silica-containing nanopaints were detectable in water and air independent of the number of abrasion cycles. Nearly 4% of tested materials were released in 160 dry abrasion cycles and the mass loss was even higher for nanocoatings upon wet abrasion (6.4%). This evidence suggests that wastewater systems could be easily “enriched” with AMC agents during their intended use alone, not considering potential active ingredient release during production and waste management phases of products life cycles. In the next chapter, the release of most widely used antimicrobial agents–silver, titanium, zinc, copper, chitosan and QACs—from AMCs are discussed. Discussion on silver-enabled AMCs is given in detail owing to the importance of silver as the most studied active ingredient in AMCs. For other AMCs ingredients, a summary of information is presented, while detailed descriptions can be found in the File S4.

#### Release of antimicrobial compounds from AMCs containing Ag, Ti, Cu, Zn, chitosan and QACs

Silver release from silver-enabled materials was reported in many papers. Out of 257 papers retrieved from the literature search (File S3), 114 (44%) contained qualitative or quantitative data on silver released from AMCs. The data were rather heterogenous in nature for the following reasons: (i) some studies were based on *in vitro* release by immersing AMCs in aqueous medium, while others reported *in vivo* release after implantation in experimental animals; (ii) release was reported either as released Ag concentration in respective test environment (e.g., g/L, mg/L, μg/mL), as Ag mass released from a surface area unit (μg/cm^2^), or as percentage of total Ag deposited on AMCs; and (iii) test conditions varied greatly by means of testing medium, temperature, pH, ionic strength, experiment duration, etc. The amount of Ag released from the AMCs depended mostly on the composition of the AMCs themselves. While some researchers deposited monolayers of elemental or Ag NPs, the majority of studies used combinations of silver with various polymers, functional groups, metal composites, antibiotics, etc. These components influence the dynamics and kinetics of silver release either due to interaction with Ag or by presenting a physical barrier to release into surrounding medium. Release of silver, specifically silver ions, from silver-containing AMCs is crucial for antimicrobial efficacy as antimicrobial effect of silver can be mostly attributed to the interaction of silver ions with components of bacterial cells (Xiu et al., 2012). Most of the reviewed papers (File S3) reported that Ag-based AMCs release Ag ions, regardless of the Ag species initially used. Ag ion release from the AMCs can be divided into two separate phases: (i) fast desorption that releases surface-bound ions and results in burst release of Ag and (ii) oxidative dissolution of Ag, which is a slower process and responsible for sustained, long-term Ag release (Gilabert-Porres et al., 2016). Both processes require a contact with surrounding medium, while oxidative dissolution additionally requires aerobic conditions. Overall, the total amount of released Ag and release kinetics depend on (i) total amount of deposited Ag, (ii) the extent of contact with the surrounding medium that can be modulated by other coating components, and (iii) the nature of deposited silver species, that is, whether coating contains atomic silver layers, embedded ions, or various forms of Ag NPs. For example, the release of embedded Ag ions is expected to be faster in the sustained-release phase, as they do not need to undergo the oxidation step. In addition, large specific surface area of Ag NPs compared to layers of atomic silver increases the contact with surrounding environment and enables faster ion release. Interestingly, two papers specifically discussed the release of Ag NPs from coated surfaces. Oćwieja, Adamczyk & Morga (2015) used poly (allylamine hydrochloride)-modified mica sheets to deposit a monolayer of Ag NPs and evaluated their desorption during 167 h. Total amount of released Ag NPs was determined by imaging of the coated surfaces before and after the release experiment. The release was reported to be fastest at lower pH and for smaller NPs. Xue et al. (2017) embedded Ag NPs in polyvinyl alcohol-polyacrylic acid polymer coated on 3D-printed implants and Ag NP release was estimated by measuring absorption. The authors stated that generally the bactericidal effect of Ag is mediated by Ag ions; presumably, the coating they developed would also release Ag ions, although this possibility was not explored.

While some researchers produced relatively simple AMCs by synthesizing an inert polymer containing Ag species, many papers investigated more complex coatings with layered architectures (Körner et al., 2010; Heidari Zare et al., 2014; Storm et al., 2015; Kuzminova et al., 2016), functional groups (Yilmaz et al., 2014; Gehring et al., 2015; He et al., 2016a), and other antimicrobial agents (Wang et al., 2016), that can improve the AMCs properties like durability, biocompatibility, responsiveness to stimuli, etc. Moreover, Ag species, especially NPs, are amenable to chemical modifications that change their functionality, interactions with other coating components, size, charge or other properties. Even though Ag release from such complex coatings followed the basic process described above, the release kinetics strongly depended on the coating architecture. We observed that general aim of these studies was to control Ag release from AMCs. Several different strategies were employed including (i) addition of polymer layers of various thickness and porosity between silver and coating surface (Heidari Zare et al., 2014; Surmeneva et al., 2017), (ii) improvement of silver adhesion to surfaces using additives (Taheri et al., 2014; Jiang et al., 2016), or (iii) deposition of Ag in contact with reducing agents, such as dopamine, to impede oxidative Ag release (Liu & Huang, 2016; Zhang et al., 2018).

According to our survey, Ag-based AMCs can be divided into two main categories:

- Coatings without barriers, that is, Ag was deposited on the surface of AMCs or impregnated into polymer matrix and no additional polymer layers or Ag-interacting additives were added.
  This group encompassed 71 papers (62%), which described the release kinetics of Ag ions. While the expected kinetics for such AMC-architectures was initial burst release followed by sustained release phase, such kinetics was only observed in coatings where Ag was deposited at or near the coating surface, or in sufficiently porous polymers that allowed water penetration. In coatings with dense polymer structure, or with hydrophobic properties, limited contact with aqueous medium reduced overall Ag release and its speed, resulting in more durable Ag release.
  Biomedical implants represent an important application of such coatings, where burst release and almost complete depletion of antimicrobial material is preferable.
- Coatings that impeded Ag release, either by covalent attachment, electrostatic interactions, control of silver species’ oxidation state, or by imposing a physical barrier, such as additional polymer layers of controlled porosity.
  In this group, 43 papers (38%) were found that employed different strategies to limit Ag release from AMC in the interest of prolonging the antibacterial effect. Several papers described coatings where Ag was encapsulated in complex structures, such as core-shells (Ho et al., 2013; Gonçalves et al., 2018), carbon nanotubes (Hao et al., 2017), etc. Another approach was to provide electrostatic interactions such as coordinating Ag ions and NPs with thiol groups (Gehring et al., 2015), while other researchers introduced functional molecules (e.g., aminosilane linker) to enable covalent binding of Ag NPs (Gomez-Carretero, Nybom & Richter-Dahlfors, 2017).

**Figure 7:**
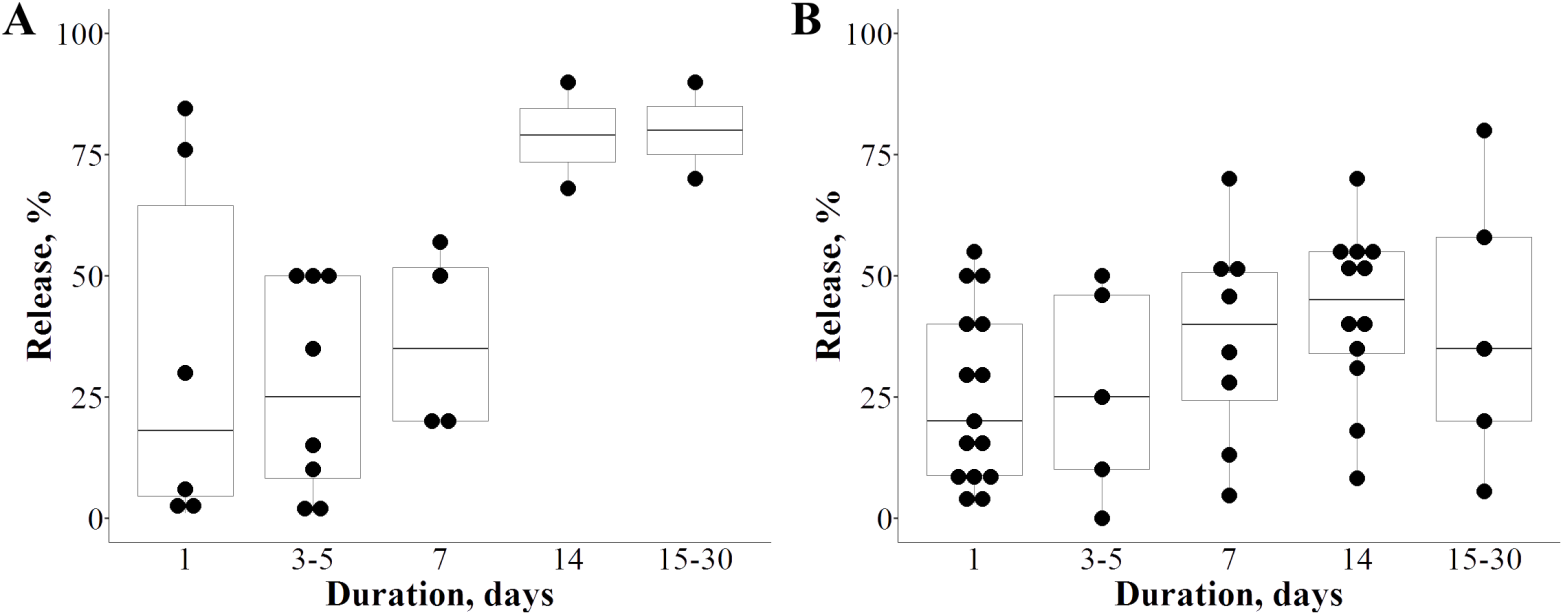
Cumulative release of silver from silver-enabled antimicrobial surface coatings in time at room temperature. (A) Uncontrolled release—no measures were taken to reduce the silver release. (B) Controlled release—additional polymer layers or other measures were taken to control the silver release. One dot represents one reported value, altogether release data from 28 papers is plotted.

In order to determine the amount of Ag that could leach into the aqueous medium from AMCs, papers containing quantitative information on the release of Ag were analyzed additionally. To achieve better comparability of available data, the analysis was performed on the subset of papers that reported Ag leaching as percentage of initial silver load (Fig. 7). Out of 114 studies that quantified silver release, 28 reports provided release data as percent of initial Ag load. Uncontrolled Ag release was reported in 11 papers, while remaining 17 papers demonstrated either controlled release or combination of controlled and uncontrolled release if different barriers for release were compared to no-barrier coating architecture. Initial Ag desorption (within first 5 days) from AMCs with no barriers was rather diverse as can be seen on Fig. 7A. Such variability was likely a result of two methodological approaches applied for Ag inclusion in the AMCs. In the first approach, architectures where Ag was deposited at the outermost layer or embedded in polymer layers near the surface presented initial burst-release followed by a more durable release. Many other studies applied second approach where embedding of Ag ions or Ag NPs within polymer was opted out causing a sustained release over the duration of experiments as Ag had no unconstrained contact with the release medium. When these two approaches were combined, the initial burst release was hindered by the sustained release data and overall leaching profile showed a relatively low cumulative release during first 5 days (median below 25%). Still, after 2 weeks or more, the cumulative release from AMCs with no barriers was similar and rather high (median ~75%) regardless of the methodology used (Fig. 7A). Interestingly, coatings with additional polymer layers or similar strategies for control of Ag release exhibited similar dissolution of silver during first week as compared to AMCs containing Ag without barriers (see Figs. 7A and 7B). However, the cumulative release data were lower for the AMCs designed for controlled release than for those without release control. Observed variability in the release data was affected by the type of strategy applied and the strength of Ag binding. Currently, it remains to be seen if coatings that utilize such release control will be used in commercial applications. Potential uses in hospitals, laboratories or other settings will eventually determine the significance of such AMCs for the environmental and ecotoxicological impacts.

For other active ingredients used in AMCs (Fig. 4) less release data was found. This may be explained by the nature of the materials and intended applications. Detailed discussion on release of titanium, copper, zinc, chitosan and QACs together with relevant references can be found from File S4.

For example, in about half of the papers on titanium-containing AMCs with release data, titanium or titanium alloys are used as a carrier for functional AMCs, especially in medical applications. Due to this, often release of antimicrobial metals (Ag, Cu, Zn, Mg), antibiotics or other antimicrobial compounds, is reported. On the other hand, Ti release is negligible due to its low solubility in aqueous media.

Cu and Zn-containing AMCs shared a common major application—marine antifouling coatings—as described in almost half of the papers providing release data. As Cu and Zn are both soluble in aqueous media and have antimicrobial properties, respective ion release was also most reported for AMCs containing either of the metals or both. Quantitative data about metal ion release was very heterogenous and impossible to generalize. Some papers also reported release of other antimicrobials such as other metal ions, organotin compounds or even nonylphenol, bisphenol A or rosin. Many of the aforementioned ingredients are specific to marine applications and aim to find alternatives to antifouling coatings falling under increasingly strict regulation such as organotin compounds. Cu- and Zn-containing AMCs degradation in time was well described for marine coatings measuring coating thickness or polishing rate leading also to the subsequent release of active ingredient or degradation product, but evaluation of such release was hard to assess quantitatively. Compared to slow-releasing marine applications, surfaces designed for medical or other specific antibacterial surfaces generally demonstrated burst release profile and high release during first hours or days of the period studied rather than stable release over weeks or months. Different cleaning protocols hold potential to greatly influence efficiency of non-invasive metal-based AMCs but were rarely investigated regarding release and depletion of active ingredient.

Similar to Ti, chitosan was mostly used as a carrier for other antimicrobial substances as an integral part of AMCs, that is, a reservoir for active substance release rather than inert substrate to accommodate AMCs. Although some papers reported results on chitosan degradation and/or possible release of degradation products, most data on release were related to antimicrobials and metal ions released from different chitosan-containing AMCs. Occasionally, release of non-antimicrobial substances was also studied. Most of the studied surfaces presented coatings with high initial burst release profile during first hours up to 1–2 days followed by period of residual slow release of variable length depending on the properties of surface material and initial loading amount of substances released from the surfaces. Strategies used to control the release of active compounds were either (i) passive via extending the release in time and/or flatten out initial burst in time by entrapping active substances using mostly layered systems and/or crosslinking or (ii) active control by pH-triggered release of antimicrobial substance. Effect of cleaning was investigated in only one paper (Mitra et al., 2017).

Quaternary ammonium compound-based AMCs differed from others as in these applications QACs were mostly covalently bound to carrier surfaces. In the papers where retention of antimicrobial activity of aged surfaces was measured, zone of inhibition of bacterial growth was assessed or leachates obtained from the AMCs evaluated for antimicrobial activity. Proof of QAC release was rarely seen. QAC-enabled AMCs were also tested for ageing/cleaning more than other AMCs discussed here but the main aim of this seemed to be evaluation of retention of antimicrobial properties of the materials and not potential QAC release. QAC release was reported from AMCs where it was not covalently bound to carrier surface, but either bound electrostatically, included in paints or degradable polymers or released as QAC-containing NPs. In two studies QAC-containing surfaces were specifically designed for triggered release of the biocidal agent.

In summary, information on release from AMCs retrieved from the literature was very heterogenous for different compounds used in various applications. Major drawback of available published data was absence of quantitative data on release, even for the surfaces where the mechanism of action is assumed to include released metal ions or other bioactive substances. Silver was the only material for which there was enough information to draw any conclusions on the release kinetics that indicated the dependence of release rate on AMCs architecture. In general, potential release of ecotoxicologically relevant amounts of Ag ions from Ag-containing AMCs could cause an adverse environmental impact. Material degradation as a reason for antimicrobials release was rarely discussed except for marine applications where degradation and/or bioactive ingredient depletion were described as changes in coating thickness, polishing rate or leached layer physical properties (i.e., by describing diminishing of the coating) and not measured as release of degradation products. The effect of cleaning on release of active ingredients or antimicrobial efficiency was also scarcely studied.

It should be highlighted that the comparability of data was generally poor due to the variability in application described, specific experimental conditions employed, and different dose metrics used to measure the release of active ingredients of AMCs. Materials for marine applications were generally incubated in artificial or natural seawater or varied salinity salt solutions with optional use-specific flow rates and temperatures, and release rate or percentage of loading amount was usually reported. AMCs meant for medical or general use were usually incubated in physiological solutions or growth medium at room or body temperature, and the results were often presented as concentrations in test medium or tissue-mimicking test system.

### Analysis of possible ecotoxicological effects of antimicrobial coatings

As mentioned above, the ecotoxicity evaluation of chemical compounds relies on tests conducted with model organisms that belong to aquatic ecosystem, such as algae, crustaceans (mostly daphnids), and fish whereas the most sensitive organism (i.e., weakest link in the ecosystem) will “rank” the compound according to its ecotoxicity (harmful, toxic, very toxic, etc.). If the concentration of a certain compound in an environmental compartment such as natural water bodies is remarkably lower than its toxic concentration to the key aquatic organisms, there will apparently be no risk to aquatic ecosystems due to this compound. In an attempt to gather information on environmental impact of antimicrobial compounds from AMCs, we analyzed papers reporting their release, but retrieved papers did not provide data on ecotoxicological effects of released compounds. Fortunately, there is already a remarkable amount of data available on the ecotoxicity of the main antimicrobial agents, that is, silver, copper, zinc, TiO_2_, QACs (Kahru & Dubourguier, 2010; Bondarenko et al., 2013; Zhang et al., 2015). The ecotoxicity data toward crustaceans, algae and fish for selected main antimicrobial ingredients currently used in AMCs is summarized in Table 1. The data were tabulated for soluble salts of silver, copper and zinc bearing in mind that the toxicity of the silver, copper and zinc-enabled AMCs is realized via respective released ions (Bondarenko et al., 2013). For QACs, ecotoxicity data for benzalkonium chloride compounds (BACs) are presented as they are among most ecotoxic QACs (Zhang et al., 2015). Also, data for soluble form of chitosan are given. Comparison of the ecotoxicity of selected AMCs ingredients (Table 1) suggested that while soluble salts of Ag, Cu and Zn, but also BACs are highly toxic to fish (respective L(E)C50 values range from 0.058 to 7.2 mg/L), their effect toward algae and crustaceans is even more severe as they are toxic to these species already at sub 0.1 μg/L level (Table 1).

**Table 1:** Acute toxicity (L(E)C _50_, mg/L) values for most often used ingredients in antimicrobial coatings toward crustaceans, algae and fish.

Group of organisms	Ag-salt	Cu-salt	Zn-salt	TiO_2_	Benzalkonium chloride compounds	Chitosan^e^
	mg metal/L			mg compound/L		
Crustaceans	0.00085^a^	0.024^a^	1.3^a^	~20,000^b^ (bulk), 67.7^b^ (nano)	0.041^c^	2.2^e^
Algae	0.0076^a^	0.007^a^	0.09^a^	60.0^b^ (bulk), 65.5^b^ (nano)	0.041^c^	3.5^e^
Fish	0.058^a^	0.28^a^	7.2^a^	500^b^ (bulk), 300^b^ (nano)	0.31^d^	3.0^e^
Lowest L(E)C_50_	0.00085	0.007	0.09	60–65.5	0.041	2.2
Most sensitive organism group	Crustaceans	Algae	Algae	Algae	Crustaceans, algae	Crustaceans

**Notes:**

a Bondarenko et al. (2013).

b Kahru & Dubourguier (2010).

c Zhang et al. (2015).

d Sigma-Aldrich (2016).

e Chitosan was dissolved in acetic acid as described in Li & Pan (2013) and analyzed for toxicity as described in Wang, Zhang & Pan (2016).

For silver-, copper- and zinc-based coating materials toxicity of respective metal ions are presented. Quaternary ammonium compounds are represented by benzalkonium chlorides as most toxic of this group according to Zhang et al. (2015).

In addition to data on ecotoxicity (dose-response information), quantitative data on exposure concentrations in surface water, sediments, soils, etc., are needed for environmental risk assessment of a certain chemical/compound. Unfortunately, the shortage of respective environmental concentrations for (emerging) environmental pollutants is the weakest link in the environmental risk assessment. According to Gottschalk, Sun & Nowack (2013), who reviewed the existing data on environmental concentrations of engineered nanomaterials (involving also TiO_2_, ZnO and Ag that are used in various AMCs), huge knowledge gaps on production, application and environmental release of nanomaterials exist. Thus, environmental risk assessment of nanomaterials is a challenging exercise and often relies on environmental concentration data based on modeling. For example, Gottschalk et al. (2009) reported following modeling-based predicted environmental concentrations for the surface waters: 0.015 μg/L for TiO_2_, NPs, 0.010 μg/L for ZnO NPs and 0.764 ng/L for Ag NPs. They concluded that only Ag NPs may cause problems for surface waters out of the analyzed compounds. Good et al. (2016) also analyzed predicted environmental concentrations of various nanomaterials (incl. TiO_2_, ZnO, CuO and Ag) in surface water concluding that these nanomaterials comprise just a small fraction of the respective background surface water concentration for a given constituent (i.e., Ti, Zn, Cu, Ag). In addition, these authors stressed that quantification of these substances is limited using current analytical methods due to their high background concentrations. For example, data on QACs concentrations in surface water are accessible due to their wide use in various cleaning products and detergents. According to Zhang et al. (2015) the three most often detected QACs in natural environments are dialkyldimethyl ammonium compounds (DADMACs, C8–C18), alkyltrimethyl ammonium compounds (ATMACs, C12–C18) and benzylalkyldimethyl ammonium compounds (BACs, C12–C18) having concentration in surface water bodies around one μg/L.

Considering the effective ecotoxic concentrations of compounds summarized in Table 1 and respective (predicted) environmental concentrations presented above, it may be concluded that the most probable AMCs ingredients to cause environmental risk are BACs followed by silver. At the same time, the environmental risk of AMCs containing TiO_2_ NPs and chitosan is rather low at current production volumes. Although the soluble chitosan was toxic to aquatic organisms at two to three mg/L (Table 1), the chitin—a precursor of chitosan, is the most abundant natural polymer and is not soluble. Both, chitin and chitosan are considered very perspective polymers for the use in biomedicine due to their biocompatibility and biodegradability (Pillai, Paul & Sharma, 2009). Concerning copper-based AMCs, increase in marine traffic and increasing amounts of copper in marine antifouling coatings possibly causing elevated local concentrations have raised concern on their effects to the environment, especially in areas considered environmentally sensitive such as the Baltic Sea or in the inland aquatic environments. Therefore, the use of copper or co-use of copper with other biocides in these applications is also regulated by law in some regions (Sweden, UK, Netherlands, Denmark) as a precautionary principle (Copper Antifouling Environmental Programme). A recent environmental assessment of nanomaterials in products listed in the Danish market suggested that NPs of CuO and Ag but also TiO_2_ NMs may also have adverse environmental impacts close to the points of discharge into the aquatic environment (Kjølholt et al., 2015).

Previous studies indicated that disinfectants pose significantly higher environmental risk than, for example, pharmaceuticals, and by far higher risk than antimicrobial agents used in AMCs as formers are still used in relatively low quantities (Orias & Perrodin, 2013; Boillot et al., 2008). Namely, in a review article by Orias & Perrodin (2013), the ecotoxicological effects of hospital effluents were studied. The results revealed that the disinfectants including free chlorine, glutaraldehyde and detergents may be present in large quantities and contribute to a great extent to the toxicity of hospital wastewaters. The quantity of detergents and disinfectants has shown to exceed by far that of pharmaceuticals in hospital effluents (Boillot et al., 2008). To get a better idea of the magnitude of disinfectant discharge from hospitals, generally, the load of disinfectants varies from two to 200 mg/L of effluent (Boillot et al., 2008). The annual consumption of glutaraldehyde is estimated to be two tons/1,000 beds per year and that of chlorine 800 kg/1,000 beds (Boillot et al., 2008). The average 24 h concentrations of detergents and disinfectants measured in hospital wastewaters include chlorine 80 mg/L, free chlorine 0.5 mg/L, non-ionic detergents 2.9 mg/L, anionic detergents <0.01 mg/L, cationic detergents five mg/L and glutaraldehyde 2.1 μg/L (Boillot et al., 2008). An estimated average annual consumption of water in hospitals varies between 109 and 657 m^3^ per bed based on different country wise and hospital wise practices (González et al., 2016).

### Conclusions, Recommendations and Outlook: focus on health-care settings

**Figure 8:**
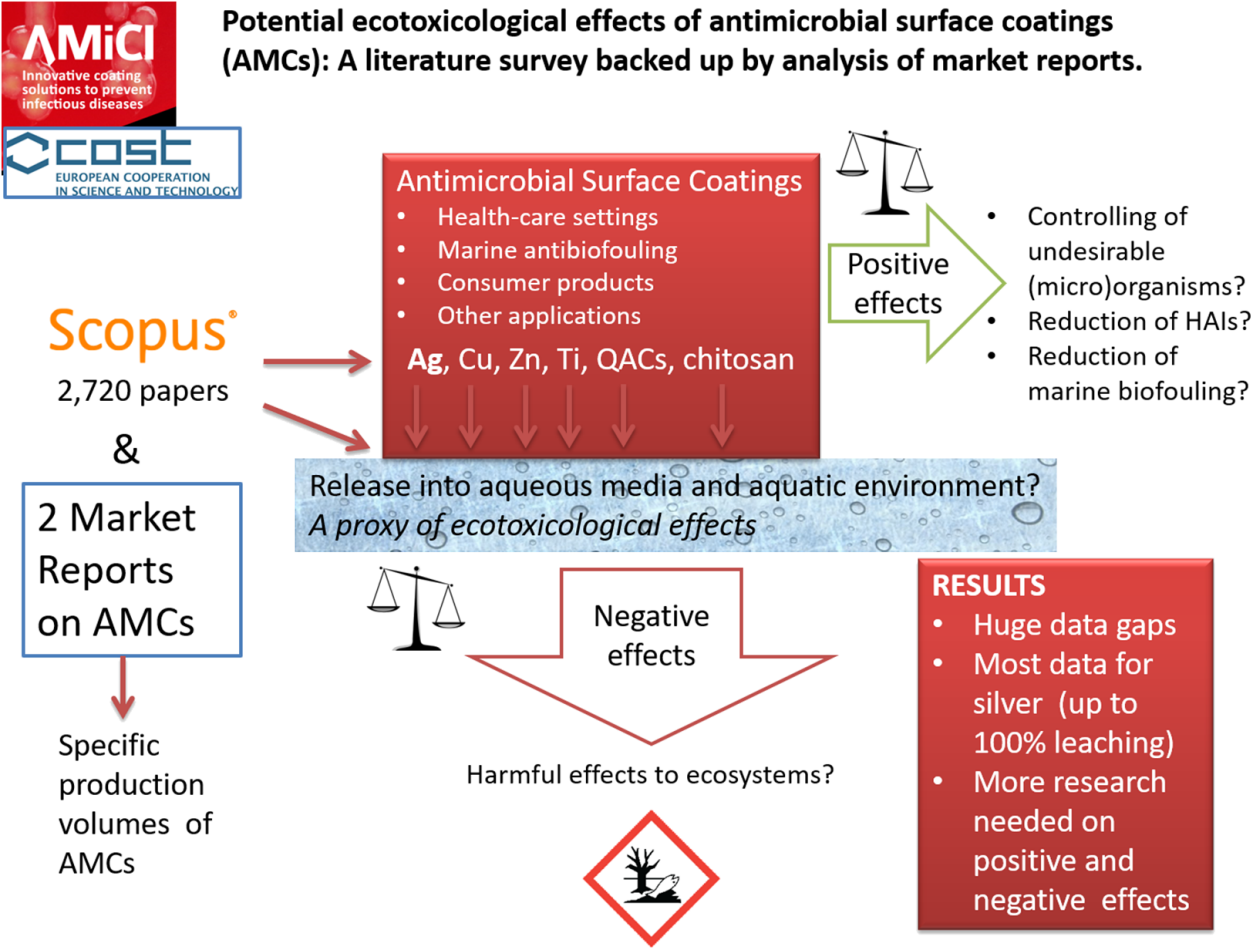
A scheme of the current review.

The current Review, as activity of the COST action CA15114 AMICI “Anti-Microbial Coating Innovations to prevent infectious diseases,” aimed to analyze the potential ecotoxicological effects of AMCs to ensure their sustainable use. For the Review, the Scopus database of scientific literature and market reports on types, application areas and production volumes of AMCs were used as data sources. The release/dissolution of the most prevalent ingredients from AMCs into the aquatic environment was used as the proxy of their possible ecotoxicological effects (Fig. 8).

The release of antimicrobial substances from AMCs is a relevant proxy also for health-care settings, as the analysis of current situation indicated that pharmaceuticals (e.g., antibiotics), heavy metals, disinfectants and detergents can be found in hospital wastewaters, in urban sewer networks and wastewater treatment plants (WWTP) (Orias & Perrodin, 2013). In addition, as WWTP processes are not usually designed or adjusted to remove these compounds, they may easily find their way to different environmental compartments resulting in potential ecotoxicity.

Based on the critical analysis of the available data from literature survey and market reports, we conclude that:

- Silver-based AMCs are by far the most studied and used active agents in AMCs. Other major ingredients of AMCs are titanium, copper, zinc, chitosan and QACs. According to retrieved literature, the main field of AMCs application is medicine followed by marine industry, paints (construction), food industry and textiles, the market reports suggest that most AMCs are used in improving the indoor air quality, which is nonetheless tightly followed by healthcare, mold remediation, construction, food industry and textiles.
- Our analysis based on market overviews provided a rough estimate on AMC production, which currently reaches hundreds of thousands of tons per year, but the variations observed in release studies do not allow us to make reliable conclusions regarding the potential concentrations of antimicrobials released to the environment.
- The literature sources indicated that the ecotoxicological aspects of antimicrobial or antifouling coatings have so far been assessed rarely and mainly in the context of marine antifouling coatings/paints. The toxicity evaluations of these coatings have mostly been performed using mammalians/mammalian cells in order to evaluate their biocompatibility.
- Only a small percentage of research articles studied and reported the release/leaching of antimicrobial ingredients. Existing information indicate that silver, copper, and zinc are often released from the coatings, while chitosan and titanium are mostly used as carriers for other antimicrobial ingredients (e.g., conventional antibiotics) which could be harmful to the environment.
- Due to the major data gaps on potential release of AMCs into different environmental compartments, starting from the aquatic ones, it is not possible to provide solid “risk-benefit” evaluation for AMCs that are used in health-care settings—one of the tasks of the EU project AMICI. Indeed, it is difficult to predict the amount of antimicrobial compounds released *specifically* from AMCs to the environment—the information that is essential for the coherent environmental risk assessment—due to the two main reasons: shortage of knowledge on the production volumes of AMCs and insufficient data on use and release of antimicrobial substances from AMCs.

As mentioned above, trace amounts of the active ingredients in AMCs like Cu, Ag or TiO_2_ may end up in the environment via hospital wastewaters due to routine cleaning practices. Hospital effluents are generally discharged into urban drainage without pre-treatment, similarly to domestic wastewater. If applied within AMCs, significant proportion of these materials could be easily released in the environment, while the non-inhibitory levels of these agents may increase the risk of developing antimicrobial resistant microbe strains. Unfortunately, the analysis of hospital wastewaters for content of silver, copper and zinc—mostly applied active agents in AMCs, is not available yet.

The analysis on ecotoxicity of silver, copper, zinc and their nanoparticulate forms (Bondarenko et al., 2013) indicated that non-target species—crustaceans and algae—are even more sensitive toward these antimicrobial compounds (see also Table 1) than microbial cells themselves. In addition to ecotoxicological effects of antimicrobials potentially released from AMCs, there is also a risk that their release in subtoxic concentrations may induce antimicrobial resistance or select for resistant strains. This happens when the concentration of antimicrobials only inhibits the growth and does not kill all of the targeted microorganisms giving an opportunity to surviving microbial population to develop resistance (Ahonen et al., 2017). This scenario has already been reported for different types of AMCs, that is, antimicrobial resistance in *Salmonella typhimurium* and *E. coli* to silver-based agents after exposure to silver-based wound dressings (Chopra, 2007), resistance to triclosan in strains of *E. coli* (Levy, 2001) and *Staphylococcus aureus* (Syed et al., 2014), or resistance to QACs in *E. coli* (Soumet et al., 2012). Moreover, such resistance may be transferred from hospital microbes to environmental microbes via mobile genetic elements (Van Hoek et al., 2011).

Lack in safety data on AMCs can obscure some undesired and even adverse effects on both human and environmental health. Many sad lessons from previous “exciting” episodes on human technological and innovation activities have been already learned, for example, the hazardous nature of asbestos that once was considered as a miracle material (Landrigan et al., 1999), or Nobel Prize award due to the discovery of dichlorodiphenyltrichloroethane that was later banned for agricultural use in the US in 1972 and restricted with Stockholm Convention on Persistent Organic Pollutants in 2001 (Yang, Ward & Kahr, 2017).

While a proper control of HAIs prevalence is necessary, a clear indication and proof for significantly higher benefits over risks of AMCs usage should be attained. More than a decade ago, the centers for disease control published guidance document with the statement that “No data support the use of these items (AMCs) as part of a sound infection-control strategy” (Sehulster et al., 2004). After 40-years long study, the US Food and Drug Administration reported in 2016 that producers failed to provide clear-cut evidences that antiseptic agents in soaps provide a benefit to human health (US Food and Drug Administration, 2016). The proper quality, efficacy and safety management of AMCs should implement Safe-by-Design (SbD) concept developed within European framework projects (e.g., NANoREG, ProSafe, NanoReg2) (Ahonen et al., 2017). The SbD concept takes into account uncertainties and potential risks of production, products and use, starting from early phase of product design. It has been already used for years by different industries like aircraft and construction industries (Hochwimmer & De Kretser, 2015). The SbD approach to development and use of AMCs balances between the efficacy of HAI prevention and the adverse impact of AMCs to both healthcare and general environment (Ahonen et al., 2017). The SbD may be implemented in responsible strategy for development and use of AMC following the recommendations of the Organization for Economic Co-operation and Development about integration of risk assessment at all stages of the life cycle of chemicals (OECD, 2015). In addition, SbD approach will help AMCs industry to align easier with the European REACH regulation (European Commission, 2006) that requires hazard assessment, exposure assessment and risk characterization of chemicals as integral parts of the chemical safety assessment process (ECHA, 2010). The hazard assessment analyzes all available and relevant data on adverse effects to both human and environmental health, including physicochemical properties, (eco)toxicological outcomes and threshold limits for occupational and environmental effects (Schimpel et al., 2018). Exposure assessment describes and identifies exposure scenarios along life-cycle of chemicals. Risk characterization estimates risk levels by combining the data on hazard and exposure. Currently there appears to be a huge data gap in the direct ecotoxicity tests of AMCs—so far only a few papers have been published in the context of ship coatings and respective (mostly marine) organisms. No studies where the potential adverse effects of AMCs were tested using a common ecotoxicological model organism *Daphnia magna*.

Thus, as recommended by Ahonen et al. (2017), the use of AMCs only on critically specified high-touch surfaces in healthcare would minimize the ecotoxicological effects considering the amounts of leaching active ingredients. Taken into account the quantities and volumes of AMC containing products compared to volumes of for example, cleaning agents and disinfectants, the related ecotoxicological risk would be marginal. The special attention needs to be taken to the cleaning procedures to avoid the potentially harmful effects to the environment. Cleaning procedures with no effluents or de-activation of effluents before discharge would enable to reduce the ecotoxicological risks for example, development of antimicrobial resistance in the environment (Dunne et al., 2018). Pre-treatment of hospital wastewaters before discharging into urban drainage would be beneficial to de-activate biocides (antibiotics, AMCs, disinfectants) and destroy the resistant microbes, and thus reduce the risk of developing antimicrobial resistance and other toxicological risks like production of organohalogen compounds due to the reactions of free chlorine.

To conclude, the risk/benefit ratio assessment on AMCs use should start already during the design phase. If the biocidal activity of the certain material or product is not obligatory/crucial in the specified indoor environment, the AMCs should not be applied.

This review reflects only the authors view.

## Supplemental Information

10.7717/peerj.6315/supp-1 Supplemental Information 1 Search phrases used in literature searches.

10.7717/peerj.6315/supp-2 Supplemental Information 2 Frequency of words and phrases related to ingredients, targets and applications of antimicrobial surface coatings in titles, abstracts and keywords of papers retrieved from Scopus.

10.7717/peerj.6315/supp-3 Supplemental Information 3 Information on release of most prevalent ingredients (silver, titanium, copper, zinc, chitosan, and quaternary ammonium compounds) from antimicrobial surface coatings.

10.7717/peerj.6315/supp-4 Supplemental Information 4 Release of antimicrobial compounds from AMCs containing Ti, Cu, Zn, chitosan, and QACs.

## Additional Information and Declarations

### Data Availability

The following information was supplied regarding data availability: The raw data for word and phrase analysis is available in Supplemental File 2.
