# Supplementary material for: Potential ecotoxicological effects of antimicrobial surface coatings: a literature survey backed up by analysis of market reports
Source: PeerJ. 2019 Feb 11;7:e6315. doi: 10.7717/peerj.6315 (PMC6375256; doi:10.7717/peerj.6315)
Supplement: Supplemental Information 1 [file peerj-07-6315-s001.doc]

**Supplemental material for**

# Potential ecotoxicological effects of antimicrobial surface coatings: A literature survey backed up by analysis of market reports.

Merilin Rosenberg ^1,2‡^, Krunoslav Ilić ^3‡^, Katre Juganson ^1^, Angela Ivask ^1^, Merja Ahonen ^4^, Ivana Vinković Vrček ^3^, Anne Kahru ^1,5^

^1^ Laboratory of Environmental Toxicology, National Institute of Chemical Physics and Biophysics, Tallinn, Estonia

^2^ Department of Chemistry and Biotechnology, Tallinn University of Technology, Tallinn, Estonia

^3^ Institute for Medical Research and Occupational Health, Zagreb, Croatia

^4^ Faculty of Technology, Satakunta University of Applied Sciences, Rauma, Finland

^5^ Estonian Academy of Sciences, Tallinn, Estonia

Corresponding Author:

Anne Kahru ^1^

Laboratory of Environmental Toxicology, National Institute of Chemical Physics and Biophysics, Akadeemia tee 23, Tallinn 12618, Estonia

Email address: [anne.kahru@kbfi.ee](mailto:anne.kahru@kbfi.ee)

^‡^ Merilin Rosenberg and Krunoslav Ilić have contributed equally to this work.

**Table S1.** Search phrases used in literature search for coverage of data on antimicrobial coatings in Scopus and Web of Science

| **Database,**  **search fields** | **Search phrase** |
| --- | --- |
| Web of Science (WoS), Topic^1^ | ((TS=("antimicrob* coat*" ) OR TS=("antimicrob* surface*" ) OR TS=("antibact* coat*" ) OR TS=("antibact* surface*") OR TS=("biocid* coat*" ) OR TS=("biocid* surface*" ) OR TS=("antibiof* coat*" ) OR TS=("antibiof* surface*" ) OR TS=("antivir* coat*" ) OR TS=("antivir* surface*" ) OR TS=("antifung* coat*" ) OR TS=("antifung* surface*" ) OR TS=("antifoul* coat*") OR TS=("antifoul* surface*" )))  *AND* **DOCUMENT TYPES:** (Article) |
| Web of Science (WoS), Title | ((TI=("antimicrob* coat*" ) OR TI=("antimicrob* surface*" ) OR TI=("antibact* coat*" ) OR TI=("antibact* surface*") OR TI=("biocid* coat*" ) OR TI=("biocid* surface*" ) OR TI=("antibiof* coat*" ) OR TI=("antibiof* surface*" ) OR TI=("antivir* coat*" ) OR TI=("antivir* surface*" ) OR TI=("antifung* coat*" ) OR TI=("antifung* surface*" ) OR TI=("antifoul* coat*") OR TI=("antifoul* surface*" )))  *AND* **DOCUMENT TYPES:** (Article) |
| Scopus,  ALL^2^ | (ALL("antimicrob* coat*") OR ALL("antimicrob* surface*") OR ALL("antibact* coat*") OR ALL("antibact* surface*") OR ALL("biocid* coat*") OR ALL("biocid* surface*") OR ALL("antibiof* coat*") OR ALL("antibiof* surface*") OR ALL("antivir* coat*") OR ALL("antivir* surface*") OR ALL("antifung* coat*") OR ALL("antifung* surface*") OR ALL("antifoul* coat*") OR ALL("antifoul* surface*")) AND DOCTYPE( ar ) |
| Scopus,  TITLE-ABS-KEY^3^ | (TITLE-ABS-KEY("antimicrob* coat*") OR TITLE‑ABS‑KEY("antimicrob* surface*") OR TITLE‑ABS‑KEY("antibact* coat*") OR TITLE-ABS-KEY("antibact* surface*") OR TITLE-ABS-KEY("biocid* coat*") OR TITLE-ABS-KEY("biocid* surface*") OR TITLE‑ABS-KEY("antibiof* coat*") OR TITLE‑ABS-KEY("antibiof* surface*") OR TITLE‑ABS-KEY("antivir* coat*") OR TITLE‑ABS-KEY("antivir* surface*") OR TITLE‑ABS-KEY("antifung* coat*") OR TITLE‑ABS-KEY("antifung* surface*") OR TITLE-ABS-KEY("antifoul* coat*") OR TITLE-ABS-KEY("antifoul* surface*")) AND DOCTYPE( ar ) |
| Scopus,  TITLE | (TITLE("antimicrob* coat*") OR TITLE("antimicrob* surface*") OR TITLE("antibact* coat*") OR TITLE("antibact* surface*") OR TITLE("biocid* coat*") OR TITLE("biocid* surface*") OR TITLE("antibiof* coat*") OR TITLE("antibiof* surface*") OR TITLE("antivir* coat*") OR TITLE("antivir* surface*") OR TITLE("antifung* coat*") OR TITLE("antifung* surface*") OR TITLE("antifoul* coat*") OR TITLE("antifoul* surface*")) AND DOCTYPE( ar ) |

^1^ Topic – performs search in the fields of Title, Abstract, Author Keywords, and Keywords Plus®
^2^ ALL – performs search in all the fields included in Scopus
^3^ TITLE-ABS-KEY – performs search in the fields of Document Title, Abstract, Author Keywords, Index Terms, Chemical Name, and Trade Name

**Table S2.** Search phrases used in literature search in Scopus database for prevalence of ingredients and evolution of information on antimicrobial surface coatings.

| **Compound** | **Search phrase** |
| --- | --- |
| total | (TITLE-ABS-KEY("antimicrob* coat*") OR TITLE‑ABS‑KEY("antimicrob* surface*") OR TITLE‑ABS‑KEY("antibact* coat*") OR TITLE-ABS-KEY("antibact* surface*") OR TITLE-ABS-KEY("biocid* coat*") OR TITLE-ABS-KEY("biocid* surface*") OR TITLE‑ABS-KEY("antibiof* coat*") OR TITLE‑ABS-KEY("antibiof* surface*") OR TITLE‑ABS-KEY("antivir* coat*") OR TITLE‑ABS-KEY("antivir* surface*") OR TITLE‑ABS-KEY("antifung* coat*") OR TITLE‑ABS-KEY("antifung* surface*") OR TITLE-ABS-KEY("antifoul* coat*") OR TITLE-ABS-KEY("antifoul* surface*")) AND DOCTYPE( ar ) |
| Ag | (TITLE-ABS-KEY("antimicrob* coat*") OR TITLE‑ABS‑KEY("antimicrob* surface*") OR TITLE‑ABS‑KEY("antibact* coat*") OR TITLE-ABS-KEY("antibact* surface*") OR TITLE-ABS-KEY("biocid* coat*") OR TITLE-ABS-KEY("biocid* surface*") OR TITLE‑ABS-KEY("antibiof* coat*") OR TITLE‑ABS-KEY("antibiof* surface*") OR TITLE‑ABS-KEY("antivir* coat*") OR TITLE‑ABS-KEY("antivir* surface*") OR TITLE‑ABS-KEY("antifung* coat*") OR TITLE‑ABS-KEY("antifung* surface*") OR TITLE-ABS-KEY("antifoul* coat*") OR TITLE-ABS-KEY("antifoul* surface*")) AND DOCTYPE( ar ) AND ( TITLE-ABS-KEY ( silver ) OR TITLE-ABS-KEY ( ag ) OR TITLE-ABS-KEY ( "AgNP*" ) OR TITLE-ABS-KEY ( nanosilver ) OR TITLE-ABS-KEY ( "nano-Ag" ) OR TITLE-ABS-KEY ( "nanoAg" ) OR TITLE-ABS-KEY ( "Ag-*" ) OR TITLE-ABS-KEY ( agno* ) OR TITLE-ABS-KEY ( agcl ) ) |
| Ag ions | (TITLE-ABS-KEY("antimicrob* coat*") OR TITLE‑ABS‑KEY("antimicrob* surface*") OR TITLE‑ABS‑KEY("antibact* coat*") OR TITLE-ABS-KEY("antibact* surface*") OR TITLE-ABS-KEY("biocid* coat*") OR TITLE-ABS-KEY("biocid* surface*") OR TITLE‑ABS-KEY("antibiof* coat*") OR TITLE‑ABS-KEY("antibiof* surface*") OR TITLE‑ABS-KEY("antivir* coat*") OR TITLE‑ABS-KEY("antivir* surface*") OR TITLE‑ABS-KEY("antifung* coat*") OR TITLE‑ABS-KEY("antifung* surface*") OR TITLE-ABS-KEY("antifoul* coat*") OR TITLE-ABS-KEY("antifoul* surface*")) AND DOCTYPE( ar ) AND ( TITLE-ABS-KEY ( "Ag ion*" ) OR TITLE-ABS-KEY ( "silver ion*" ) OR TITLE-ABS-KEY ( "silver nitrate" ) OR TITLE-ABS-KEY ( agno* ) OR TITLE-ABS-KEY ( agcl ) ) AND NOT ( TITLE-ABS-KEY ( "silver nano*" ) OR TITLE-ABS-KEY ( "Ag NP*" ) OR TITLE-ABS-KEY ( "AgNP*" ) OR TITLE-ABS-KEY ( "Ag nano*" ) ) |
| Ag NPs | ((TITLE-ABS-KEY(“antimicrob* coat*”) OR TITLE‑ABS‑KEY(“antimicrob* surface*”) OR TITLE‑ABS‑KEY(“antibact* coat*”) OR TITLE-ABS-KEY(“antibact* surface*”) OR TITLE-ABS-KEY(“biocid* coat*”) OR TITLE-ABS-KEY(“biocid* surface*”) OR TITLE‑ABS-KEY(“antibiof* coat*”) OR TITLE‑ABS-KEY(“antibiof* surface*”) OR TITLE‑ABS-KEY(“antivir* coat*”) OR TITLE‑ABS-KEY(“antivir* surface*”) OR TITLE‑ABS-KEY(“antifung* coat*”) OR TITLE‑ABS-KEY(“antifung* surface*”) OR TITLE-ABS-KEY(“antifoul* coat*”) OR TITLE-ABS-KEY(“antifoul* surface*”)) AND DOCTYPE( ar ) AND ( TITLE-ABS-KEY ( “silver nano*” ) OR TITLE-ABS-KEY ( “Ag NP*” ) OR TITLE-ABS-KEY ( “AgNP*” ) OR TITLE-ABS-KEY ( “Ag nano*” ) ) |
| antibiotic | (TITLE-ABS-KEY("antimicrob* coat*") OR TITLE‑ABS‑KEY("antimicrob* surface*") OR TITLE‑ABS‑KEY("antibact* coat*") OR TITLE-ABS-KEY("antibact* surface*") OR TITLE-ABS-KEY("biocid* coat*") OR TITLE-ABS-KEY("biocid* surface*") OR TITLE‑ABS-KEY("antibiof* coat*") OR TITLE‑ABS-KEY("antibiof* surface*") OR TITLE‑ABS-KEY("antivir* coat*") OR TITLE‑ABS-KEY("antivir* surface*") OR TITLE‑ABS-KEY("antifung* coat*") OR TITLE‑ABS-KEY("antifung* surface*") OR TITLE-ABS-KEY("antifoul* coat*") OR TITLE-ABS-KEY("antifoul* surface*")) AND DOCTYPE( ar ) AND ( TITLE-ABS-KEY ( antibiotic ) ) |
| chitosan | (TITLE-ABS-KEY(“antimicrob* coat*”) OR TITLE‑ABS‑KEY(“antimicrob* surface*”) OR TITLE‑ABS‑KEY(“antibact* coat*”) OR TITLE-ABS-KEY(“antibact* surface*”) OR TITLE-ABS-KEY(“biocid* coat*”) OR TITLE-ABS-KEY(“biocid* surface*”) OR TITLE‑ABS-KEY(“antibiof* coat*”) OR TITLE‑ABS-KEY(“antibiof* surface*”) OR TITLE‑ABS-KEY(“antivir* coat*”) OR TITLE‑ABS-KEY(“antivir* surface*”) OR TITLE‑ABS-KEY(“antifung* coat*”) OR TITLE‑ABS-KEY(“antifung* surface*”) OR TITLE-ABS-KEY(“antifoul* coat*”) OR TITLE-ABS-KEY(“antifoul* surface*”)) AND DOCTYPE( ar ) AND ( TITLE-ABS-KEY ( chitosan ) ) |
| copolymers | (TITLE-ABS-KEY("antimicrob* coat*") OR TITLE‑ABS‑KEY("antimicrob* surface*") OR TITLE‑ABS‑KEY("antibact* coat*") OR TITLE-ABS-KEY("antibact* surface*") OR TITLE-ABS-KEY("biocid* coat*") OR TITLE-ABS-KEY("biocid* surface*") OR TITLE‑ABS-KEY("antibiof* coat*") OR TITLE‑ABS-KEY("antibiof* surface*") OR TITLE‑ABS-KEY("antivir* coat*") OR TITLE‑ABS-KEY("antivir* surface*") OR TITLE‑ABS-KEY("antifung* coat*") OR TITLE‑ABS-KEY("antifung* surface*") OR TITLE-ABS-KEY("antifoul* coat*") OR TITLE-ABS-KEY("antifoul* surface*")) AND DOCTYPE( ar ) AND (TITLE-ABS-KEY ( copolymer* ) ) |
| Cu | (TITLE-ABS-KEY(“antimicrob* coat*”) OR TITLE‑ABS‑KEY(“antimicrob* surface*”) OR TITLE‑ABS‑KEY(“antibact* coat*”) OR TITLE-ABS-KEY(“antibact* surface*”) OR TITLE-ABS-KEY(“biocid* coat*”) OR TITLE-ABS-KEY(“biocid* surface*”) OR TITLE‑ABS-KEY(“antibiof* coat*”) OR TITLE‑ABS-KEY(“antibiof* surface*”) OR TITLE‑ABS-KEY(“antivir* coat*”) OR TITLE‑ABS-KEY(“antivir* surface*”) OR TITLE‑ABS-KEY(“antifung* coat*”) OR TITLE‑ABS-KEY(“antifung* surface*”) OR TITLE-ABS-KEY(“antifoul* coat*”) OR TITLE-ABS-KEY(“antifoul* surface*”)) AND DOCTYPE( ar ) AND ( TITLE-ABS-KEY ( “copper*” ) OR TITLE-ABS-KEY ( cu ) OR TITLE-ABS-KEY ( cuo ) ) |
| CuO_x_ | TITLE-ABS-KEY("antimicrob* coat*") OR TITLE‑ABS‑KEY("antimicrob* surface*") OR TITLE‑ABS‑KEY("antibact* coat*") OR TITLE-ABS-KEY("antibact* surface*") OR TITLE-ABS-KEY("biocid* coat*") OR TITLE-ABS-KEY("biocid* surface*") OR TITLE‑ABS-KEY("antibiof* coat*") OR TITLE‑ABS-KEY("antibiof* surface*") OR TITLE‑ABS-KEY("antivir* coat*") OR TITLE‑ABS-KEY("antivir* surface*") OR TITLE‑ABS-KEY("antifung* coat*") OR TITLE‑ABS-KEY("antifung* surface*") OR TITLE-ABS-KEY("antifoul* coat*") OR TITLE-ABS-KEY("antifoul* surface*")) AND DOCTYPE( ar ) AND ( TITLE-ABS-KEY ( "copper ox*" ) OR TITLE-ABS-KEY ( cuo ) ) |
| metal | (TITLE-ABS-KEY("antimicrob* coat*") OR TITLE‑ABS‑KEY("antimicrob* surface*") OR TITLE‑ABS‑KEY("antibact* coat*") OR TITLE-ABS-KEY("antibact* surface*") OR TITLE-ABS-KEY("biocid* coat*") OR TITLE-ABS-KEY("biocid* surface*") OR TITLE‑ABS-KEY("antibiof* coat*") OR TITLE‑ABS-KEY("antibiof* surface*") OR TITLE‑ABS-KEY("antivir* coat*") OR TITLE‑ABS-KEY("antivir* surface*") OR TITLE‑ABS-KEY("antifung* coat*") OR TITLE‑ABS-KEY("antifung* surface*") OR TITLE-ABS-KEY("antifoul* coat*") OR TITLE-ABS-KEY("antifoul* surface*")) AND DOCTYPE( ar ) AND ( TITLE-ABS-KEY ( metal ) ) |
| quaternary ammonium compounds | (TITLE-ABS-KEY("antimicrob* coat*") OR TITLE‑ABS‑KEY("antimicrob* surface*") OR TITLE‑ABS‑KEY("antibact* coat*") OR TITLE-ABS-KEY("antibact* surface*") OR TITLE-ABS-KEY("biocid* coat*") OR TITLE-ABS-KEY("biocid* surface*") OR TITLE‑ABS-KEY("antibiof* coat*") OR TITLE‑ABS-KEY("antibiof* surface*") OR TITLE‑ABS-KEY("antivir* coat*") OR TITLE‑ABS-KEY("antivir* surface*") OR TITLE‑ABS-KEY("antifung* coat*") OR TITLE‑ABS-KEY("antifung* surface*") OR TITLE-ABS-KEY("antifoul* coat*") OR TITLE-ABS-KEY("antifoul* surface*")) AND DOCTYPE( ar ) AND ( TITLE-ABS-KEY ( "quaternary ammonium" ) ) |
| peptides | (TITLE-ABS-KEY("antimicrob* coat*") OR TITLE‑ABS‑KEY("antimicrob* surface*") OR TITLE‑ABS‑KEY("antibact* coat*") OR TITLE-ABS-KEY("antibact* surface*") OR TITLE-ABS-KEY("biocid* coat*") OR TITLE-ABS-KEY("biocid* surface*") OR TITLE‑ABS-KEY("antibiof* coat*") OR TITLE‑ABS-KEY("antibiof* surface*") OR TITLE‑ABS-KEY("antivir* coat*") OR TITLE‑ABS-KEY("antivir* surface*") OR TITLE‑ABS-KEY("antifung* coat*") OR TITLE‑ABS-KEY("antifung* surface*") OR TITLE-ABS-KEY("antifoul* coat*") OR TITLE-ABS-KEY("antifoul* surface*")) AND DOCTYPE( ar ) AND ( TITLE-ABS-KEY ( peptide* ) ) |
| polyethylene glycol (PEG) | (TITLE-ABS-KEY("antimicrob* coat*") OR TITLE‑ABS‑KEY("antimicrob* surface*") OR TITLE‑ABS‑KEY("antibact* coat*") OR TITLE-ABS-KEY("antibact* surface*") OR TITLE-ABS-KEY("biocid* coat*") OR TITLE-ABS-KEY("biocid* surface*") OR TITLE‑ABS-KEY("antibiof* coat*") OR TITLE‑ABS-KEY("antibiof* surface*") OR TITLE‑ABS-KEY("antivir* coat*") OR TITLE‑ABS-KEY("antivir* surface*") OR TITLE‑ABS-KEY("antifung* coat*") OR TITLE‑ABS-KEY("antifung* surface*") OR TITLE-ABS-KEY("antifoul* coat*") OR TITLE-ABS-KEY("antifoul* surface*")) AND DOCTYPE( ar ) AND ( TITLE-ABS-KEY ( peg ) OR TITLE-ABS-KEY ( "polyethylene glycol*" ) ) |
| silica | (TITLE-ABS-KEY("antimicrob* coat*") OR TITLE‑ABS‑KEY("antimicrob* surface*") OR TITLE‑ABS‑KEY("antibact* coat*") OR TITLE-ABS-KEY("antibact* surface*") OR TITLE-ABS-KEY("biocid* coat*") OR TITLE-ABS-KEY("biocid* surface*") OR TITLE‑ABS-KEY("antibiof* coat*") OR TITLE‑ABS-KEY("antibiof* surface*") OR TITLE‑ABS-KEY("antivir* coat*") OR TITLE‑ABS-KEY("antivir* surface*") OR TITLE‑ABS-KEY("antifung* coat*") OR TITLE‑ABS-KEY("antifung* surface*") OR TITLE-ABS-KEY("antifoul* coat*") OR TITLE-ABS-KEY("antifoul* surface*")) AND DOCTYPE( ar ) AND ( TITLE-ABS-KEY ( silica ) ) |
| silicon | (TITLE-ABS-KEY("antimicrob* coat*") OR TITLE‑ABS‑KEY("antimicrob* surface*") OR TITLE‑ABS‑KEY("antibact* coat*") OR TITLE-ABS-KEY("antibact* surface*") OR TITLE-ABS-KEY("biocid* coat*") OR TITLE-ABS-KEY("biocid* surface*") OR TITLE‑ABS-KEY("antibiof* coat*") OR TITLE‑ABS-KEY("antibiof* surface*") OR TITLE‑ABS-KEY("antivir* coat*") OR TITLE‑ABS-KEY("antivir* surface*") OR TITLE‑ABS-KEY("antifung* coat*") OR TITLE‑ABS-KEY("antifung* surface*") OR TITLE-ABS-KEY("antifoul* coat*") OR TITLE-ABS-KEY("antifoul* surface*")) AND DOCTYPE( ar ) AND ( TITLE-ABS-KEY ( silicon ) ) |
| silicone | (TITLE-ABS-KEY("antimicrob* coat*") OR TITLE‑ABS‑KEY("antimicrob* surface*") OR TITLE‑ABS‑KEY("antibact* coat*") OR TITLE-ABS-KEY("antibact* surface*") OR TITLE-ABS-KEY("biocid* coat*") OR TITLE-ABS-KEY("biocid* surface*") OR TITLE‑ABS-KEY("antibiof* coat*") OR TITLE‑ABS-KEY("antibiof* surface*") OR TITLE‑ABS-KEY("antivir* coat*") OR TITLE‑ABS-KEY("antivir* surface*") OR TITLE‑ABS-KEY("antifung* coat*") OR TITLE‑ABS-KEY("antifung* surface*") OR TITLE-ABS-KEY("antifoul* coat*") OR TITLE-ABS-KEY("antifoul* surface*")) AND DOCTYPE( ar ) AND ( TITLE-ABS-KEY ( silicone ) ) |
| stainless | (TITLE-ABS-KEY("antimicrob* coat*") OR TITLE‑ABS‑KEY("antimicrob* surface*") OR TITLE‑ABS‑KEY("antibact* coat*") OR TITLE-ABS-KEY("antibact* surface*") OR TITLE-ABS-KEY("biocid* coat*") OR TITLE-ABS-KEY("biocid* surface*") OR TITLE‑ABS-KEY("antibiof* coat*") OR TITLE‑ABS-KEY("antibiof* surface*") OR TITLE‑ABS-KEY("antivir* coat*") OR TITLE‑ABS-KEY("antivir* surface*") OR TITLE‑ABS-KEY("antifung* coat*") OR TITLE‑ABS-KEY("antifung* surface*") OR TITLE-ABS-KEY("antifoul* coat*") OR TITLE-ABS-KEY("antifoul* surface*")) AND DOCTYPE( ar ) AND ( TITLE-ABS-KEY ( stainless* ) ) |
| Ti | (TITLE-ABS-KEY("antimicrob* coat*") OR TITLE‑ABS‑KEY("antimicrob* surface*") OR TITLE‑ABS‑KEY("antibact* coat*") OR TITLE-ABS-KEY("antibact* surface*") OR TITLE-ABS-KEY("biocid* coat*") OR TITLE-ABS-KEY("biocid* surface*") OR TITLE‑ABS-KEY("antibiof* coat*") OR TITLE‑ABS-KEY("antibiof* surface*") OR TITLE‑ABS-KEY("antivir* coat*") OR TITLE‑ABS-KEY("antivir* surface*") OR TITLE‑ABS-KEY("antifung* coat*") OR TITLE‑ABS-KEY("antifung* surface*") OR TITLE-ABS-KEY("antifoul* coat*") OR TITLE-ABS-KEY("antifoul* surface*")) AND DOCTYPE( ar ) AND ( TITLE-ABS-KEY ( titanium ) OR TITLE-ABS-KEY ( ti ) OR TITLE-ABS-KEY ( tio2 ) OR TITLE-ABS-KEY ( titania ) ) |
| TiO_2_ | (TITLE-ABS-KEY("antimicrob* coat*") OR TITLE‑ABS‑KEY("antimicrob* surface*") OR TITLE‑ABS‑KEY("antibact* coat*") OR TITLE-ABS-KEY("antibact* surface*") OR TITLE-ABS-KEY("biocid* coat*") OR TITLE-ABS-KEY("biocid* surface*") OR TITLE‑ABS-KEY("antibiof* coat*") OR TITLE‑ABS-KEY("antibiof* surface*") OR TITLE‑ABS-KEY("antivir* coat*") OR TITLE‑ABS-KEY("antivir* surface*") OR TITLE‑ABS-KEY("antifung* coat*") OR TITLE‑ABS-KEY("antifung* surface*") OR TITLE-ABS-KEY("antifoul* coat*") OR TITLE-ABS-KEY("antifoul* surface*")) AND DOCTYPE( ar ) AND ( TITLE-ABS-KEY ( "titanium diox*" ) OR TITLE-ABS-KEY ( tio2 ) OR TITLE-ABS-KEY ( titania ) ) |
| Zn | (TITLE-ABS-KEY("antimicrob* coat*") OR TITLE‑ABS‑KEY("antimicrob* surface*") OR TITLE‑ABS‑KEY("antibact* coat*") OR TITLE-ABS-KEY("antibact* surface*") OR TITLE-ABS-KEY("biocid* coat*") OR TITLE-ABS-KEY("biocid* surface*") OR TITLE‑ABS-KEY("antibiof* coat*") OR TITLE‑ABS-KEY("antibiof* surface*") OR TITLE‑ABS-KEY("antivir* coat*") OR TITLE‑ABS-KEY("antivir* surface*") OR TITLE‑ABS-KEY("antifung* coat*") OR TITLE‑ABS-KEY("antifung* surface*") OR TITLE-ABS-KEY("antifoul* coat*") OR TITLE-ABS-KEY("antifoul* surface*")) AND DOCTYPE( ar ) AND ( TITLE-ABS-KEY ( zinc ) OR TITLE-ABS-KEY ( zn ) OR TITLE-ABS-KEY ( zno ) ) |
| ZnO | (TITLE-ABS-KEY("antimicrob* coat*") OR TITLE‑ABS‑KEY("antimicrob* surface*") OR TITLE‑ABS‑KEY("antibact* coat*") OR TITLE-ABS-KEY("antibact* surface*") OR TITLE-ABS-KEY("biocid* coat*") OR TITLE-ABS-KEY("biocid* surface*") OR TITLE‑ABS-KEY("antibiof* coat*") OR TITLE‑ABS-KEY("antibiof* surface*") OR TITLE‑ABS-KEY("antivir* coat*") OR TITLE‑ABS-KEY("antivir* surface*") OR TITLE‑ABS-KEY("antifung* coat*") OR TITLE‑ABS-KEY("antifung* surface*") OR TITLE-ABS-KEY("antifoul* coat*") OR TITLE-ABS-KEY("antifoul* surface*")) AND DOCTYPE( ar ) AND ( TITLE-ABS-KEY ( "zinc ox*" ) OR TITLE-ABS-KEY ( zno ) ) |

**Table S3.** Search phrases used in literature search in Scopus database for selection of papers discussing environment-related aspects.

| **Phrase retrieved by phrase analysis of Title-ABS-KEY of total amount of papers** | **Search phrase** |
| --- | --- |
| environmentally friendly | (TITLE-ABS-KEY("antimicrob* coat*") OR TITLE‑ABS‑KEY("antimicrob* surface*") OR TITLE‑ABS‑KEY("antibact* coat*") OR TITLE-ABS-KEY("antibact* surface*") OR TITLE-ABS-KEY("biocid* coat*") OR TITLE-ABS-KEY("biocid* surface*") OR TITLE‑ABS-KEY("antibiof* coat*") OR TITLE‑ABS-KEY("antibiof* surface*") OR TITLE‑ABS-KEY("antivir* coat*") OR TITLE‑ABS-KEY("antivir* surface*") OR TITLE‑ABS-KEY("antifung* coat*") OR TITLE‑ABS-KEY("antifung* surface*") OR TITLE-ABS-KEY("antifoul* coat*") OR TITLE-ABS-KEY("antifoul* surface*")) AND DOCTYPE( ar ) AND ( TITLE-ABS-KEY ( "environmentally friendly")) |
| environmentally benign | (TITLE-ABS-KEY("antimicrob* coat*") OR TITLE‑ABS‑KEY("antimicrob* surface*") OR TITLE‑ABS‑KEY("antibact* coat*") OR TITLE-ABS-KEY("antibact* surface*") OR TITLE-ABS-KEY("biocid* coat*") OR TITLE-ABS-KEY("biocid* surface*") OR TITLE‑ABS-KEY("antibiof* coat*") OR TITLE‑ABS-KEY("antibiof* surface*") OR TITLE‑ABS-KEY("antivir* coat*") OR TITLE‑ABS-KEY("antivir* surface*") OR TITLE‑ABS-KEY("antifung* coat*") OR TITLE‑ABS-KEY("antifung* surface*") OR TITLE-ABS-KEY("antifoul* coat*") OR TITLE-ABS-KEY("antifoul* surface*")) AND DOCTYPE( ar ) AND ( TITLE-ABS-KEY ( "environmentally benign")) |
| environmental impact | (TITLE-ABS-KEY("antimicrob* coat*") OR TITLE‑ABS‑KEY("antimicrob* surface*") OR TITLE‑ABS‑KEY("antibact* coat*") OR TITLE-ABS-KEY("antibact* surface*") OR TITLE-ABS-KEY("biocid* coat*") OR TITLE-ABS-KEY("biocid* surface*") OR TITLE‑ABS-KEY("antibiof* coat*") OR TITLE‑ABS-KEY("antibiof* surface*") OR TITLE‑ABS-KEY("antivir* coat*") OR TITLE‑ABS-KEY("antivir* surface*") OR TITLE‑ABS-KEY("antifung* coat*") OR TITLE‑ABS-KEY("antifung* surface*") OR TITLE-ABS-KEY("antifoul* coat*") OR TITLE-ABS-KEY("antifoul* surface*")) AND DOCTYPE( ar ) AND ( TITLE-ABS-KEY ( "environmental impact")) |
| low toxicity | (TITLE-ABS-KEY("antimicrob* coat*") OR TITLE‑ABS‑KEY("antimicrob* surface*") OR TITLE‑ABS‑KEY("antibact* coat*") OR TITLE-ABS-KEY("antibact* surface*") OR TITLE-ABS-KEY("biocid* coat*") OR TITLE-ABS-KEY("biocid* surface*") OR TITLE‑ABS-KEY("antibiof* coat*") OR TITLE‑ABS-KEY("antibiof* surface*") OR TITLE‑ABS-KEY("antivir* coat*") OR TITLE‑ABS-KEY("antivir* surface*") OR TITLE‑ABS-KEY("antifung* coat*") OR TITLE‑ABS-KEY("antifung* surface*") OR TITLE-ABS-KEY("antifoul* coat*") OR TITLE-ABS-KEY("antifoul* surface*")) AND DOCTYPE( ar ) AND ( TITLE-ABS-KEY ( "low toxicity")) |
