# Supplementary material for: Potential ecotoxicological effects of antimicrobial surface coatings: a literature survey backed up by analysis of market reports
Source: PeerJ. 2019 Feb 11;7:e6315. doi: 10.7717/peerj.6315 (PMC6375256; doi:10.7717/peerj.6315)
Supplement: Supplemental Information 4 [file peerj-07-6315-s004.docx]

**Supplemental material for**

# Potential ecotoxicological effects of antimicrobial surface coatings: A literature survey backed up by analysis of market reports.

Merilin Rosenberg ^1,2‡^, Krunoslav Ilić ^3‡^, Katre Juganson ^1^, Angela Ivask ^1^, Merja Ahonen ^4^, Ivana Vinković Vrček ^3^, Anne Kahru ^1,5^

^1^ Laboratory of Environmental Toxicology, National Institute of Chemical Physics and Biophysics, Tallinn, Estonia

^2^ Department of Chemistry and Biotechnology, Tallinn University of Technology, Tallinn, Estonia

^3^ Institute for Medical Research and Occupational Health, Zagreb, Croatia

^4^ Faculty of Technology, Satakunta University of Applied Sciences, Rauma, Finland

^5^ Estonian Academy of Sciences, Tallinn, Estonia

Corresponding Author:

Anne Kahru ^1^

Laboratory of Environmental Toxicology, National Institute of Chemical Physics and Biophysics, Akadeemia tee 23, Tallinn 12618, Estonia

Email address: [anne.kahru@kbfi.ee](mailto:anne.kahru@kbfi.ee)

^‡^ Merilin Rosenberg and Krunoslav Ilić have contributed equally to this work.

## Referenced results from the literature survey for Ti, Cu, Zn, chitosan and QAC containing surfaces

### *Titanium containing coatings*

For titanium-based AMCs, 165 papers were retrieved with the “release” search. However, 55% of these papers concerned materials that were deposited on titanium or titanium alloy substrates. Out of papers where titanium was used as an antimicrobial ingredient, only four (2.4%) studied the amount of released Ti: no titanium was released from stainless steel intramedullary nails coated with titanium oxide and siloxane polymer doped with Ag neodecanoate into goat serum samples (Gladitz, Reinemann & Radusch, 2009), no leaching of titanium ion from the hetero-nanostructured multilayer films comprising negatively charged titania nanosheets and positively charged lysozyme into phosphate-buffered saline was observed (Wang & Zhang, 2012), Ti release was negligible from Ti6Al4V substrates and the same substrates coated with a thin film of Si/F (Santos-Coquillat et al., 2018), and only Godoy-Gallardo et al. (2016) reported trace amounts of titanium in tissues around dental implants. More often the release of other substances from Ti substrates or Ti-containing coatings was studied. For example, 34 papers, included in the previous chapter where silver had been used together with titanium, the release of Ag ions was studied. In coatings where a combination of titanium with other metals was used, release of Cu (Finke et al., 2012; Wei et al., 2014a; Peres et al., 2014; Hempel et al., 2014; Chen et al., 2014a), Zn (Huang et al., 2017), and Mg (Zaatreh et al., 2017) was also reported. Titanium coatings have been also combined with antibiotics or other antimicrobials where release of vancomycin (Swanson, Cheng & Friedrich, 2011; Pitarresi et al., 2013; Wang et al., 2016; Han et al., 2017; Bakhshandeh et al., 2017), gentamicin (Moskowitz et al., 2010; Pérez-Anes et al., 2015; Hizal et al., 2015; Wang et al., 2017b), minocycline (Darouiche, 2007; Lv et al., 2014), rifampin (Kälicke et al., 2006; Darouiche, 2007), tetracycline (Cai et al., 2016; Bottino et al., 2017), ciprofloxacin (Mattioli-Belmonte et al., 2014) and fusidic acid (Kälicke et al., 2006); antiseptics like chlorhexidine (Harris et al., 2006; Chang et al., 2014; Wood et al., 2015), octenidin and irgasan (Kälicke et al., 2006); antimicrobial peptides like catestatin (Özçelik et al., 2015), recombinant human b-defensin-2 (rHubD2)(Pfeufer et al., 2011), Cationic Steroidal Antimicrobial‐13 (CSA‐13) (Sinclair et al., 2012), cov-GL13K (derived from the human salivary protein Parotid Secretory Protein (BPIFA2)) (Chen et al., 2014b); or other molecules like cyclodextrin (Pérez-Anes et al., 2015) and butenolide (Chen, Xia & Qian, 2017), was studied.

### *Copper containing coatings*

Release from copper-containing AMCs was mentioned in 122 documents, with only 36 results (30%) containing information on release of some substances. Among these, 32 papers had data on copper release. Besides copper, also (co-)release of other substances that were used in combination with copper was investigated from copper-containing surfaces: silver (Yeasmin et al., 2016; Harrasser et al., 2016; Haase et al., 2017; Ciacotich et al., 2018), zinc (Watermann et al., 2005; Sathya et al., 2016; Ytreberg et al., 2017), lead (Watermann et al., 2005), chromium (Watermann et al., 2005), cadmium (Watermann et al., 2005), nickel (Watermann et al., 2005), organotin compounds (Thomas et al., 1999; Watermann et al., 2005), hydrogen peroxide (Olsen et al., 2010), diuron (Faÿ et al., 2018), tolylfluanid (Faÿ et al., 2018), copper thiocyanate (Faÿ et al., 2018), 4,5-dichloro-2-n-octyl-3(2H)-isothiazolone (Sea Nine®[Rohm Haas]) (Howell & Behrends, 2006), nonylphenol (Watermann et al., 2005), bisphenol A (Watermann et al., 2005), rosin (Peres et al., 2014). Marine (15 of 36 papers, 42%) and medical/general purpose (18 of 36 papers, 50%) applications were more or less equally represented, when special applications such as reverse osmosis membranes (Ben-Sasson et al., 2014), water filtration systems (Padmavathy et al., 2017) or photo-excited outdoor applications (Wei et al., 2014a) were not accounted for. Influence of cleaning on active ingredient release and degradation of coatings has been rarely studied. Namely, only two cases were found for copper-containing AMCs: abrasive cleaning of marine coating (Schiff, Diehl & Valkirs, 2004) and repeatedly wiping copper-loaded chitosan/silica surfaces with water (Mitra et al., 2017). Degradation of the surface was studied in one paper as rosin dissolution in non-marine application (Wei et al., 2014b).

Copper release rate from "Lacomit"lacquered pure copper surface was about 160 μg/cm2 per day after 2 weeks and 70 μg/cm2 per day after 18 weeks of static incubation in seawater and less for copper alloys (Hall & Baker, 1985, 1986). For copper-based marine antifouling coatings, either commercial or experimental, stable release rates after a few weeks were from under 1 μg/cm2 per day (~4 weeks (Sathya et al., 2016)) to ~20-30 μg/cm2 per day in different periods (2.5 weeks (Thomas et al., 1999); 4 weeks (Howell & Behrends, 2006); ~12 weeks (Valkirs et al., 2003)). As for silver-based AMCs, also in copper containing AMCs, copper release was experimentally lowered for example by using Cu nanowires in elastomeric polydimethylsiloxane (PDMS) (stable release ~35 ng/cm2 per day after ~1.5 weeks and less than 2 % of total copper on the surface was released during 50 days (Jiang et al., 2015)) or by exploiting using hydrophobic nanocomposite films (Sathya et al., 2016). Control strategies to prolong the release also included crosslinking (Padmavathy et al., 2017) or encapsulation of active ingredients (Liu et al., 2017). Abrasive cleaning of surfaces painted with commercial antifouling paints containing cuprous oxide increased short-term release rate for about 3-fold for a few days after which the release dropped to baseline long-term release rate (Schiff, Diehl & Valkirs, 2004). Compared to slow-releasing marine applications, surfaces with proposed medical or other specific or general antibacterial surface application generally demonstrated burst release profile and high release for first hours or days of the experiment rather than stable release for weeks or months. For example, Taglietti et al. (2018) showed total Cu depletion at physiological pH already in 5 hours, Ben-Sasson et al. (2014) demonstrated 30% Cu depletion during 24 h from reverse osmosis membranes, and Rubin, Neufeld & Reynolds (2018) reported 54-90% Cu depletion from textile during 24 h. Different cleaning protocols potentially greatly influence efficiency of non-invasive AMCs but are rarely investigated regarding release and depletion of active ingredient. For example, wiping copper-loaded acrylated quaternized chitosan/silica on poly(vinyl fluoride) films with deionized water moistened paper resulted in no decrease to 67% decrease in Cu content after 60 wipes and 68%-90% decrease in Cu content after 100 wipes, depending on specific surface architecture (Mitra et al., 2017).

### *Zinc containing coatings*

Zinc, in the context of release from AMCs was mentioned in 66 articles with 27 papers (41%) containing at least some kind of data on release. Among these, only 17 had (semi‑)quantitative data for zinc release. Besides zinc, also (co-)release of other substances used in combination with zinc was investigated from zinc-containing surfaces: silver (Coughlan et al., 2008; Agnihotri et al., 2015; Kumar S. Mural et al., 2016), copper (Watermann et al., 2005; Sathya et al., 2016; Ytreberg et al., 2017), hydrogen peroxide (Olsen et al., 2009; Kristensen et al., 2010), crystal violet (Noimark et al., 2015; Ozkan, Allan & Parkin, 2018), paeonolsilate (Sun et al., 2016; Yang et al., 2017), salicylate (Bellotti et al., 2013; Bellotti & Romagnoli, 2014), magnesium (Zaatreh et al., 2017), vanadium (Zhou et al., 2016), lead (Watermann et al., 2005), chromium (Watermann et al., 2005), cadmium (Watermann et al., 2005), nickel (Watermann et al., 2005), organotin compounds (Watermann et al., 2005), chlorine dioxide (Li et al., 2009), chlorhexidine (Carteau et al., 2014), butenolide (Chen, Xia & Qian, 2017), benzoate (Bellotti et al., 2013), polyphenols (Bellotti, del Amo & Romagnoli, 2012a), chitosan (Al-Naamani et al., 2017), nonylphenol (Watermann et al., 2005), bisphenol A (Watermann et al., 2005). Cleaning or reusing the surfaces was generally not explored with a couple of exceptions e.g., washing ZnO NP-containing textile (Manna et al., 2013) or repeated use of Ag/ZnO nanomaterial-coated surfaces (Agnihotri et al., 2015). Material degradation was rarely evaluated except for marine applications where degradation is described as thickness or polishing rate of the coating and not measured as release of degradation products. Marine antifouling coating was proposed application in 13 (48%) of the 27 studies including specific material architecture and release study conditions with stable long-term releases up to 100 μg/cm2 per day (Mu et al., 2017). Less than half of the papers proposed medical applications, from invasive device coatings to more general surfaces that could be used in healthcare settings or other public areas. Surfaces for medical applications generally demonstrated burst release profile with for example 85-96% Zn depletion in 24 h (Malzahn et al., 2013). Slower release rates were achieved by using zinc-incorporated chitosan/gelatin nanocomposite coatings (10-20% Zn loss in 4 weeks) (Huang et al., 2017).

### *Chitosan containing coatings*

Release from chitosan-based AMCs was documented in 74 papers, out of which 26 results (35%) contained actual release data. Among these, in 6 papers chitosan degradation and/or possible release of degradation products was evaluated (Huang et al., 2011; Avetta et al., 2014; Pérez-Anes et al., 2015; Ma et al., 2015; Mitra et al., 2016; Al-Naamani et al., 2017). In most studies (12 papers), chitosan was used as a reservoir for other antimicrobials: vancomycin (Swanson, Cheng & Friedrich, 2011; Bakhshandeh et al., 2017), gentamicin (Ma et al., 2015; Xu et al., 2017), ciprofloxacin (Avetta et al., 2014; Mattioli-Belmonte et al., 2014), tetracycline (Cai et al., 2016), cefepime (Pebdeni, Sadri & Pebdeni, 2016), minocycline (Lv et al., 2014), β-peptide (Raman et al., 2016), berberine (Huang et al., 2011), triclosan (Wang et al., 2017a). In 10 papers ionic metal release was recorded: silver (Pang & Zhitomirsky, 2008; Travan et al., 2011; Li et al., 2013a; Nie et al., 2017; Bakhshandeh et al., 2017; Cometa et al., 2017; Guo et al., 2018); copper (Abiraman & Balasubramanian, 2017; Mitra et al., 2017); zinc (Huang et al., 2017). In a few cases, also non-antimicrobials e.g., toluidine blue ortho (TBO) (Pérez-Anes et al., 2015), bovine serum albumin (BSA) (Ma et al., 2015), anticancer drug doxorubicin (Ma et al., 2015) or liquid smoke (Ceylan et al., 2017) were incorporated into chitosan surface coatings. Effect of cleaning was investigated in only one paper (Mitra et al., 2017).

Most of the studied surfaces presented coatings with high initial burst release profile during first hours up to 1-2 days of release experiment followed by period of residual slow release of variable length depending on surface material properties and initial loading amount of substances released from the surfaces. Strategies used to control the release of active compounds were either passive via extending the release in time and/or flatten out initial burst in time by entrapping active substances using mostly layered systems and/or crosslinking (Pang & Zhitomirsky, 2008; Lv et al., 2014; Pérez-Anes et al., 2015; Ma et al., 2015; Raman et al., 2016; Mitra et al., 2016; Nie et al., 2017; Xu et al., 2017) or active control by pH-triggered release of antimicrobial substance (Wang et al., 2017a).

### *Coatings containing quaternary ammonium compounds*

57 articles mentioned QACs in the context of release from AMCs to which QACs were either covalently crosslinked (33 articles), deposited (6 articles), mixed (2 articles), grafted, immobilized, impregnated (all 1 article), or introduced by another method. In 18 articles, release of QACs from surfaces was not discussed at all, while 20 (35%) articles out of 57 contained certain indicative information that could be used to assess the stability of surfaces. For example, Liu et al. (2015), He et al. (2016), Yan et al. (2017), Sugii et al. (2017), and Cuervo-Rodríguez et al. (2017) assumed no potential QAC leakage. Their conclusion was based on the chemical properties of the used QACs or due to their covalent linking with the surfaces. Another group of articles (Yang et al., 2012; Farah et al., 2013; Michailidis et al., 2017) tested the ability (and thus, indirectly also the leakage of QACs) of surfaces to retain their antimicrobial properties after storage e.g., in seawater (Yang et al., 2012; Michailidis et al., 2017), phosphate-buffered saline (Beyth et al., 2008), deionized water (Church et al., 2017), bacterial growth media (Saini et al., 2016) or detergent-containing water (Saif et al., 2015) after specified storage time ranging from weeks till 5 months, or after significant number of washes using detergent-containing water (e.g., QAC-containing textiles in Saif et al. (2015)). In all these studies, except in the Church et al. (2017), antimicrobial effect of coatings was retained during storage or after washing, and no sign of its decrease was observed. Only Church et al. (2017) observed release of QAC-containing NPs from antimicrobial concrete surface whereas the proof for QAC NP (Fixed Quat) release was antimicrobial activity of wash water. In addition to testing of the antimicrobial activity of aged QAC surfaces, some articles also evaluated leaching of QACs by using zone of inhibition (ZOI) test (Lee et al., 2004; Kurt et al., 2007; Beyth et al., 2008; Yagci et al., 2011a,b; Asri et al., 2014; He et al., 2015; Zhao et al., 2015). In these assessments, the QAC containing surfaces were placed as small discs onto agarized surface (rich growth medium or blood containing agar on which the microbial suspension was spread) and zone of inhibition of bacterial growth around the discs was analyzed. In none of these papers ZOI was observed, i.e. no leakage of QACs was concluded. Another group of papers investigated the stability of QAC surfaces by leaching the surfaces and subsequently testing the antimicrobial effects of leachates (Han et al., 2011; Jellali et al., 2013; Asri et al., 2014; Church et al., 2017). In none of these studies, the tested leachate exhibited antimicrobial effect. Only 6 (11%) articles out of 57 measured the release of QACs or quantified the QAC release. Chen et al. (2017) followed the amount of chlorine as a proxy for QAC content on surfaces. They observed that during single leaching with 0.15% AATCC detergent solution, 65% of antibacterial components were removed, while removal was 67% or 80% with 3 and 5 washing cycles, respectively. Such a decrease of antibacterial components did not render the surfaces ineffective as even after 10 washing cycles the surfaces stayed antibacterial (as 0.05 wt% QAC, a threshold antibacterial concentration, was still present on the surfaces). Pérez-Köhler et al. (2016) used HPLC to follow chlorhexidine release from polypropylene surfaces. From these surfaces, to which the QAC was bound electrostatically, 20% of chlorhexidine was released during first hour, ~30% was released during 5 h, ~ 40% during 24 h, ~55% during 48 h, and 100% of chlorhexidine was released during 72 h incubation in phosphate buffered saline. In another study, Bellotti, del Amo & Romagnoli (2012b) demonstrated the QAC release from paints based on rosin/oleic acid and rosin/polystyrene by analyzing the amount of QAC-associated polyphenols. In seawater, from a surface with initial content of 1.5 g QAC/cm2, 1-3.5 µg/cm2 (thus, 0.0001-0.0003% of the initial material) was released in one day. A study by Coneski, Fulmer & Wynne (2012) quantitatively assessed the hydrolysis of degradable polymer to which quaternary ammonium salt was added. In distilled water, 8-25% of polymer was degraded within 4 weeks and 10-35% of the polymer within 16 weeks. In two studies (Wei et al., 2016, 2017), QAC containing surfaces were specifically designed for triggered release of the biocidal agent. In the earlier study, Wei et al. (2016) demonstrated destruction of β-cyclodextrin attached to surfaces via electrostatic forces by use of surfactants such as SDS. Indeed, most of the β-cyclodextrin was removed from surfaces after SDS treatment. Later, Wei et al. (2017) used UV light at 365 nm 10 mW/cm2 to trigger the QAC release and demonstrated that 30 min of exposure to UV was enough to release 89.6% of the used azobenzene-quaternary ammonium salt complex.

## References

Abiraman T., Balasubramanian S. 2017. Synthesis and Characterization of Large-Scale (<2 nm) Chitosan-Decorated Copper Nanoparticles and Their Application in Antifouling Coating. *Industrial & Engineering Chemistry Research* 56:1498–1508. DOI: 10.1021/acs.iecr.6b04692.

Agnihotri S., Bajaj G., Mukherji S., Mukherji S. 2015. Arginine-assisted immobilization of silver nanoparticles on ZnO nanorods: an enhanced and reusable antibacterial substrate without human cell cytotoxicity. *Nanoscale* 7:7415–7429. DOI: 10.1039/C4NR06913G.

Al-Naamani L., Dobretsov S., Dutta J., Burgess JG. 2017. Chitosan-zinc oxide nanocomposite coatings for the prevention of marine biofouling. *Chemosphere* 168:408–417. DOI: 10.1016/j.chemosphere.2016.10.033.

Asri LATW., Crismaru M., Roest S., Chen Y., Ivashenko O., Rudolf P., Tiller JC., van der Mei HC., Loontjens TJA., Busscher HJ. 2014. A Shape-Adaptive, Antibacterial-Coating of Immobilized Quaternary-Ammonium Compounds Tethered on Hyperbranched Polyurea and its Mechanism of Action. *Advanced Functional Materials* 24:346–355. DOI: 10.1002/adfm.201301686.

Avetta P., Nisticò R., Faga MG., D’Angelo D., Boot EA., Lamberti R., Martorana S., Calza P., Fabbri D., Magnacca G. 2014. Hernia-repair prosthetic devices functionalised with chitosan and ciprofloxacin coating: controlled release and antibacterial activity. *Journal of Materials Chemistry B* 2:5287. DOI: 10.1039/C4TB00236A.

Bakhshandeh S., Gorgin Karaji Z., Lietaert K., Fluit AC., Boel CHE., Vogely HC., Vermonden T., Hennink WE., Weinans H., Zadpoor AA., Amin Yavari S. 2017. Simultaneous Delivery of Multiple Antibacterial Agents from Additively Manufactured Porous Biomaterials to Fully Eradicate Planktonic and Adherent *Staphylococcus aureus*. *ACS Applied Materials & Interfaces* 9:25691–25699. DOI: 10.1021/acsami.7b04950.

Bellotti N., del Amo B., Romagnoli R. 2012a. Tara tannin a natural product with antifouling coating application. *Progress in Organic Coatings* 74:411–417. DOI: 10.1016/j.porgcoat.2011.11.014.

Bellotti N., del Amo B., Romagnoli R. 2012b. Quaternary Ammonium “Tannate” for Antifouling Coatings. *Industrial & Engineering Chemistry Research* 51:16626–16632. DOI: 10.1021/ie301524r.

Bellotti N., Romagnoli R. 2014. Assessment of Zinc Salicylate as Antifouling Product for Marine Coatings. *Industrial & Engineering Chemistry Research* 53:14559–14564. DOI: 10.1021/ie5015734.

Bellotti N., Salvatore L., Deyá C., Del Panno MT., del Amo B., Romagnoli R. 2013. The application of bioactive compounds from the food industry to control mold growth in indoor waterborne coatings. *Colloids and Surfaces B: Biointerfaces* 104:140–144. DOI: 10.1016/j.colsurfb.2012.11.037.

Ben-Sasson M., Zodrow KR., Genggeng Q., Kang Y., Giannelis EP., Elimelech M. 2014. Surface Functionalization of Thin-Film Composite Membranes with Copper Nanoparticles for Antimicrobial Surface Properties. *Environmental Science & Technology* 48:384–393. DOI: 10.1021/es404232s.

Beyth N., Houri-Haddad Y., Baraness-Hadar L., Yudovin-Farber I., Domb AJ., Weiss EI. 2008. Surface antimicrobial activity and biocompatibility of incorporated polyethylenimine nanoparticles. *Biomaterials* 29:4157–4163. DOI: 10.1016/j.biomaterials.2008.07.003.

Bottino MC., Münchow EA., Albuquerque MTP., Kamocki K., Shahi R., Gregory RL., Chu T-MG., Pankajakshan D. 2017. Tetracycline-incorporated polymer nanofibers as a potential dental implant surface modifier: TETRACYCLINE-INCORPORATED POLYMER NANOFIBERS. *Journal of Biomedical Materials Research Part B: Applied Biomaterials* 105:2085–2092. DOI: 10.1002/jbm.b.33743.

Cai X., Ma K., Zhou Y., Jiang T., Wang Y. 2016. Surface functionalization of titanium with tetracycline loaded chitosan–gelatin nanosphere coatings via EPD: fabrication, characterization and mechanism. *RSC Advances* 6:7674–7682. DOI: 10.1039/C5RA17109A.

Carteau D., Vallée-Réhel K., Linossier I., Quiniou F., Davy R., Compère C., Delbury M., Faÿ F. 2014. Development of environmentally friendly antifouling paints using biodegradable polymer and lower toxic substances. *Progress in Organic Coatings* 77:485–493. DOI: 10.1016/j.porgcoat.2013.11.012.

Ceylan Z., Unal Sengor GF., Sağdıç O., Yilmaz MT. 2017. A novel approach to extend microbiological stability of sea bass ( Dicentrarchus labrax ) fillets coated with electrospun chitosan nanofibers. *LWT - Food Science and Technology* 79:367–375. DOI: 10.1016/j.lwt.2017.01.062.

Chang C-H., Yeh S-Y., Lee B-H., Hsu C-W., Chen Y-C., Chen C-J., Lin T-J., Hung-Chih Chen M., Huang C-T., Chen H-Y. 2014. Compatibility balanced antibacterial modification based on vapor-deposited parylene coatings for biomaterials. *J. Mater. Chem. B* 2:8496–8503. DOI: 10.1039/C4TB00992D.

Chen S., Guo Y., Zhong H., Chen S., Li J., Ge Z., Tang J. 2014a. Synergistic antibacterial mechanism and coating application of copper/titanium dioxide nanoparticles. *Chemical Engineering Journal* 256:238–246. DOI: 10.1016/j.cej.2014.07.006.

Chen X., Hirt H., Li Y., Gorr S-U., Aparicio C. 2014b. Antimicrobial GL13K Peptide Coatings Killed and Ruptured the Wall of Streptococcus gordonii and Prevented Formation and Growth of Biofilms. *PLoS ONE* 9:e111579. DOI: 10.1371/journal.pone.0111579.

Chen L., Xia C., Qian P-Y. 2017. Optimization of antifouling coatings incorporating butenolide, a potent antifouling agent via field and laboratory tests. *Progress in Organic Coatings* 109:22–29. DOI: 10.1016/j.porgcoat.2017.04.014.

Chen Y., Yu P., Feng C., Wang Y., Han Q., Zhang Q. 2017. Synthesis of polysiloxane with quaternized N-halamine moieties for antibacterial coating of polypropylene via supercritical impregnation technique. *Applied Surface Science* 419:683–691. DOI: 10.1016/j.apsusc.2017.05.087.

Church J., Kannan H., An J., Lee WH., Santra S., Nam BH. 2017. Recycled concrete aggregate coated with quaternary ammonium compounds for water disinfection. *International Journal of Environmental Science and Technology* 14:543–552. DOI: 10.1007/s13762-016-1168-z.

Ciacotich N., Din RU., Sloth JJ., Møller P., Gram L. 2018. An electroplated copper–silver alloy as antibacterial coating on stainless steel. *Surface and Coatings Technology* 345:96–104. DOI: 10.1016/j.surfcoat.2018.04.007.

Cometa S., Bonifacio MA., Baruzzi F., de Candia S., Giangregorio MM., Giannossa LC., Dicarlo M., Mattioli-Belmonte M., Sabbatini L., De Giglio E. 2017. Silver-loaded chitosan coating as an integrated approach to face titanium implant-associated infections: analytical characterization and biological activity. *Analytical and Bioanalytical Chemistry* 409:7211–7221. DOI: 10.1007/s00216-017-0685-z.

Coneski PN., Fulmer PA., Wynne JH. 2012. Thermal polycondensation of poly(diol citrate)s with tethered quaternary ammonium biocides. *RSC Advances* 2:12824. DOI: 10.1039/c2ra21286b.

Coughlan A., Boyd D., Douglas CWI., Towler MR. 2008. Antibacterial coatings for medical devices based on glass polyalkenoate cement chemistry. *Journal of Materials Science: Materials in Medicine* 19:3555–3560. DOI: 10.1007/s10856-008-3519-x.

Cuervo-Rodríguez R., López-Fabal F., Gómez-Garcés JL., Muñoz-Bonilla A., Fernández-García M. 2017. Contact Active Antimicrobial Coatings Prepared by Polymer Blending. *Macromolecular Bioscience* 17:1700258. DOI: 10.1002/mabi.201700258.

Darouiche RO. 2007. In Vivo Efficacy of Antimicrobial-Coated Devices. *The Journal of Bone and Joint Surgery (American)* 89:792. DOI: 10.2106/JBJS.F.00414.

Farah S., Khan W., Farber I., Kesler-Shvero D., Beyth N., Weiss EI., Domb AJ. 2013. Crosslinked QA-PEI nanoparticles: synthesis reproducibility, chemical modifications, and stability study: CROSSLINKED QA-PEI NANOPARTICLES. *Polymers for Advanced Technologies* 24:446–452. DOI: 10.1002/pat.3102.

Faÿ F., Horel G., Linossier I., Vallée-Réhel K. 2018. Effect of biocidal coatings on microfouling: In vitro and in situ results. *Progress in Organic Coatings* 114:162–172. DOI: 10.1016/j.porgcoat.2017.10.017.

Finke B., Polak M., Hempel F., Rebl H., Zietz C., Stranak V., Lukowski G., Hippler R., Bader R., Nebe JB., Weltmann K-D., Schröder K. 2012. Antimicrobial Potential of Copper-Containing Titanium Surfaces Generated by Ion Implantation and Dual High Power Impulse Magnetron Sputtering. *Advanced Engineering Materials* 14:B224–B230. DOI: 10.1002/adem.201180054.

Gladitz M., Reinemann S., Radusch H-J. 2009. Preparation of Silver Nanoparticle Dispersions via a Dendritic-Polymer Template Approach and their Use for Antibacterial Surface Treatment. *Macromolecular Materials and Engineering* 294:178–189. DOI: 10.1002/mame.200800269.

Godoy-Gallardo M., Manzanares-Céspedes MC., Sevilla P., Nart J., Manzanares N., Manero JM., Gil FJ., Boyd SK., Rodríguez D. 2016. Evaluation of bone loss in antibacterial coated dental implants: An experimental study in dogs. *Materials Science and Engineering: C* 69:538–545. DOI: 10.1016/j.msec.2016.07.020.

Guo R., Wen J., Gao Y., Li T., Yan H., Wang H., Niu B., Jiang K. 2018. Effect of the adhesion of Ag coatings on the effectiveness and durability of antibacterial properties. *Journal of Materials Science* 53:4759–4767. DOI: 10.1007/s10853-017-1939-z.

Haase H., Jordan L., Keitel L., Keil C., Mahltig B. 2017. Comparison of methods for determining the effectiveness of antibacterial functionalized textiles. *PLOS ONE* 12:e0188304. DOI: 10.1371/journal.pone.0188304.

Hall A., Baker AJM. 1985. Settlement and growth of copper-tolerantEctocarpus siliculosus (Dillw.) Lyngbye on different copper-based antifouling surfaces under laboratory conditions: Part 1 Corrosion trials in seawater and development of an algal culture system. *Journal of Materials Science* 20:1111–1118. DOI: 10.1007/BF00585756.

Hall A., Baker AJM. 1986. Settlement and growth of copper-tolerantEctocarpus siliculosus (Dillw.) Lyngbye on different copper-based antifouling surfaces under laboratory conditions: Part 2 A comparison of the early stages of fouling using light and scanning electron microscopy. *Journal of Materials Science* 21:1240–1252. DOI: 10.1007/BF00553258.

Han H., Wu J., Avery CW., Mizutani M., Jiang X., Kamigaito M., Chen Z., Xi C., Kuroda K. 2011. Immobilization of Amphiphilic Polycations by Catechol Functionality for Antimicrobial Coatings. *Langmuir* 27:4010–4019. DOI: 10.1021/la1046904.

Han J., Yang Y., Lu J., Wang C., Xie Y., Zheng X., Yao Z., Zhang C. 2017. Sustained release vancomycin-coated titanium alloy using a novel electrostatic dry powder coating technique may be a potential strategy to reduce implant-related infection. *BioScience Trends* 11:346–354. DOI: 10.5582/bst.2017.01061.

Harrasser N., Jüssen S., Obermeir A., Kmeth R., Stritzker B., Gollwitzer H., Burgkart R. 2016. Antibacterial potency of different deposition methods of silver and copper containing diamond-like carbon coated polyethylene. *Biomaterials Research* 20. DOI: 10.1186/s40824-016-0062-6.

Harris LG., Mead L., Müller-Oberländer E., Richards RG. 2006. Bacteria and cell cytocompatibility studies on coated medical grade titanium surfaces. *Journal of Biomedical Materials Research Part A* 78A:50–58. DOI: 10.1002/jbm.a.30611.

He W., Zhang Y., Li J., Gao Y., Luo F., Tan H., Wang K., Fu Q. 2016. A Novel Surface Structure Consisting of Contact-active Antibacterial Upper-layer and Antifouling Sub-layer Derived from Gemini Quaternary Ammonium Salt Polyurethanes. *Scientific Reports* 6. DOI: 10.1038/srep32140.

He W., Zhang Y., Luo F., Li J., Wang K., Tan H., Fu Q. 2015. A novel non-releasing antibacterial poly(styrene-acrylate)/waterborne polyurethane composite containing gemini quaternary ammonium salt. *RSC Advances* 5:89763–89770. DOI: 10.1039/C5RA16714K.

Hempel F., Finke B., Zietz C., Bader R., Weltmann K-D., Polak M. 2014. Antimicrobial surface modification of titanium substrates by means of plasma immersion ion implantation and deposition of copper. *Surface and Coatings Technology* 256:52–58. DOI: 10.1016/j.surfcoat.2014.01.027.

Hizal F., Zhuk I., Sukhishvili S., Busscher HJ., van der Mei HC., Choi C-H. 2015. Impact of 3D Hierarchical Nanostructures on the Antibacterial Efficacy of a Bacteria-Triggered Self-Defensive Antibiotic Coating. *ACS Applied Materials & Interfaces* 7:20304–20313. DOI: 10.1021/acsami.5b05947.

Howell D., Behrends B. 2006. A methodology for evaluating biocide release rate, surface roughness and leach layer formation in a TBT-free, self-polishing antifouling coating. *Biofouling* 22:303–315. DOI: 10.1080/08927010600924304.

Huang P., Ma K., Cai X., Huang D., Yang X., Ran J., Wang F., Jiang T. 2017. Enhanced antibacterial activity and biocompatibility of zinc-incorporated organic-inorganic nanocomposite coatings via electrophoretic deposition. *Colloids and Surfaces B: Biointerfaces* 160:628–638. DOI: 10.1016/j.colsurfb.2017.10.012.

Huang D., Zuo Y., Zou Q., Zhang L., Li J., Cheng L., Shen J., Li Y. 2011. Antibacterial Chitosan Coating on Nano-hydroxyapatite/Polyamide66 Porous Bone Scaffold for Drug Delivery. *Journal of Biomaterials Science, Polymer Edition* 22:931–944. DOI: 10.1163/092050610X496576.

Jellali R., Campistron I., Pasetto P., Laguerre A., Gohier F., Hellio C., Pilard J-F., Mouget J-L. 2013. Antifouling activity of novel polyisoprene-based coatings made from photocurable natural rubber derived oligomers. *Progress in Organic Coatings* 76:1203–1214. DOI: 10.1016/j.porgcoat.2013.03.028.

Jiang S., Sreethawong T., Lee SSC., Low MBJ., Win KY., Brzozowska AM., Teo SL-M., Vancso GJ., Jańczewski D., Han M-Y. 2015. Fabrication of Copper Nanowire Films and their Incorporation into Polymer Matrices for Antibacterial and Marine Antifouling Applications. *Advanced Materials Interfaces* 2:1400483. DOI: 10.1002/admi.201400483.

Kälicke T., Schierholz J., Schlegel U., Frangen TM., Köller M., Printzen G., Seybold D., Klöckner S., Muhr G., Arens S. 2006. Effect on infection resistance of a local antiseptic and antibiotic coating on osteosynthesis implants: An in vitro and in vivo study. *Journal of Orthopaedic Research* 24:1622–1640. DOI: 10.1002/jor.20193.

Kristensen JB., Meyer RL., Poulsen CH., Kragh KM., Besenbacher F., Laursen BS. 2010. Biomimetic silica encapsulation of enzymes for replacement of biocides in antifouling coatings. *Green Chem.* 12:387–394. DOI: 10.1039/B913772F.

Kumar S. Mural P., Jain S., Madras G., Bose S. 2016. Improving antifouling ability by site-specific silver decoration on polyethylene ionomer membranes for water remediation: assessed using 3D micro computed tomography, water flux and antibacterial studies. *RSC Advances* 6:88057–88065. DOI: 10.1039/C6RA18043D.

Kurt P., Wood L., Ohman DE., Wynne KJ. 2007. Highly Effective Contact Antimicrobial Surfaces via Polymer Surface Modifiers. *Langmuir* 23:4719–4723. DOI: 10.1021/la063718m.

Lee SB., Koepsel RR., Morley SW., Matyjaszewski K., Sun Y., Russell AJ. 2004. Permanent, Nonleaching Antibacterial Surfaces. 1. Synthesis by Atom Transfer Radical Polymerization. *Biomacromolecules* 5:877–882. DOI: 10.1021/bm034352k.

Li Y., Leung WK., Yeung KL., Lau PS., Kwan JKC. 2009. A Multilevel Antimicrobial Coating Based on Polymer-Encapsulated ClO _2_. *Langmuir* 25:13472–13480. DOI: 10.1021/la901974d.

Li P., Zhang X., Xu R., Wang W., Liu X., Yeung KWK., Chu PK. 2013a. Electrochemically deposited chitosan/Ag complex coatings on biomedical NiTi alloy for antibacterial application. *Surface and Coatings Technology* 232:370–375. DOI: 10.1016/j.surfcoat.2013.05.037.

Liu Y., Suo X., Wang Z., Gong Y., Wang X., Li H. 2017. Developing polyimide-copper antifouling coatings with capsule structures for sustainable release of copper. *Materials & Design* 130:285–293. DOI: 10.1016/j.matdes.2017.05.075.

Liu R., Zheng J., Li Z., Liu J., Liu X. 2015. Preparation of surface self-concentration and contact-killing antibacterial coating through UV curing. *RSC Advances* 5:34199–34205. DOI: 10.1039/C5RA03881B.

Lv H., Chen Z., Yang X., Cen L., Zhang X., Gao P. 2014. Layer-by-layer self-assembly of minocycline-loaded chitosan/alginate multilayer on titanium substrates to inhibit biofilm formation. *Journal of Dentistry* 42:1464–1472. DOI: 10.1016/j.jdent.2014.06.003.

Ma L., Cheng C., He C., Nie C., Deng J., Sun S., Zhao C. 2015. Substrate-Independent Robust and Heparin-Mimetic Hydrogel Thin Film Coating via Combined LbL Self-Assembly and Mussel-Inspired Post-Cross-linking. *ACS Applied Materials & Interfaces* 7:26050–26062. DOI: 10.1021/acsami.5b09634.

Malzahn K., Duque L., Ciernak P., Wiesenmüller S., Bender K., Förch R. 2013. Antimicrobial Activity and Cyto-Compatibility of Plasma Polymerized Zinc Acetylacetonate Thin Films. *Plasma Processes and Polymers* 10:243–249. DOI: 10.1002/ppap.201200106.

Manna J., Begum G., Kumar KP., Misra S., Rana RK. 2013. Enabling Antibacterial Coating via Bioinspired Mineralization of Nanostructured ZnO on Fabrics under Mild Conditions. *ACS Applied Materials & Interfaces* 5:4457–4463. DOI: 10.1021/am400933n.

Mattioli-Belmonte M., Cometa S., Ferretti C., Iatta R., Trapani A., Ceci E., Falconi M., De Giglio E. 2014. Characterization and cytocompatibility of an antibiotic/chitosan/cyclodextrins nanocoating on titanium implants. *Carbohydrate Polymers* 110:173–182. DOI: 10.1016/j.carbpol.2014.03.097.

Michailidis M., Sorzabal-Bellido I., Adamidou EA., Diaz-Fernandez YA., Aveyard J., Wengier R., Grigoriev D., Raval R., Benayahu Y., D’Sa RA., Shchukin D. 2017. Modified Mesoporous Silica Nanoparticles with a Dual Synergetic Antibacterial Effect. *ACS Applied Materials & Interfaces* 9:38364–38372. DOI: 10.1021/acsami.7b14642.

Mitra D., Li M., Kang E-T., Neoh KG. 2017. Transparent Copper-Loaded Chitosan/Silica Antibacterial Coatings with Long-Term Efficacy. *ACS Applied Materials & Interfaces* 9:29515–29525. DOI: 10.1021/acsami.7b07700.

Mitra D., Li M., Wang R., Tang Z., Kang E-T., Neoh KG. 2016. Scalable Aqueous-Based Process for Coating Polymer and Metal Substrates with Stable Quaternized Chitosan Antibacterial Coatings. *Industrial & Engineering Chemistry Research* 55:9603–9613. DOI: 10.1021/acs.iecr.6b02201.

Moskowitz JS., Blaisse MR., Samuel RE., Hsu H-P., Harris MB., Martin SD., Lee JC., Spector M., Hammond PT. 2010. The effectiveness of the controlled release of gentamicin from polyelectrolyte multilayers in the treatment of Staphylococcus aureus infection in a rabbit bone model. *Biomaterials* 31:6019–6030. DOI: 10.1016/j.biomaterials.2010.04.011.

Mu Y., Wu Z., Ma Y., Zheng J., Zhang W., Sun Z., Wang X., Pei D., Li L., Jiang W., Hou J., Wan X. 2017. Robust mussel-inspired coatings for controlled zinc ion release. *Journal of Materials Chemistry B* 5:1742–1752. DOI: 10.1039/C6TB03176E.

Nie C., Yang Y., Cheng C., Ma L., Deng J., Wang L., Zhao C. 2017. Bioinspired and biocompatible carbon nanotube-Ag nanohybrid coatings for robust antibacterial applications. *Acta Biomaterialia* 51:479–494. DOI: 10.1016/j.actbio.2017.01.027.

Noimark S., Weiner J., Noor N., Allan E., Williams CK., Shaffer MSP., Parkin IP. 2015. Dual-Mechanism Antimicrobial Polymer-ZnO Nanoparticle and Crystal Violet-Encapsulated Silicone. *Advanced Functional Materials* 25:1367–1373. DOI: 10.1002/adfm.201402980.

Olsen SM., Kristensen JB., Laursen BS., Pedersen LT., Dam-Johansen K., Kiil S. 2010. Antifouling effect of hydrogen peroxide release from enzymatic marine coatings: Exposure testing under equatorial and Mediterranean conditions. *Progress in Organic Coatings* 68:248–257. DOI: 10.1016/j.porgcoat.2010.01.003.

Olsen SM., Pedersen LT., Hermann MH., Kiil S., Dam-Johansen K. 2009. Inorganic precursor peroxides for antifouling coatings. *Journal of Coatings Technology and Research* 6:187–199. DOI: 10.1007/s11998-008-9143-3.

Özçelik H., Vrana NE., Gudima A., Riabov V., Gratchev A., Haikel Y., Metz-Boutigue M-H., Carradò A., Faerber J., Roland T., Klüter H., Kzhyshkowska J., Schaaf P., Lavalle P. 2015. Harnessing the Multifunctionality in Nature: A Bioactive Agent Release System with Self-Antimicrobial and Immunomodulatory Properties. *Advanced Healthcare Materials* 4:2026–2036. DOI: 10.1002/adhm.201500546.

Ozkan E., Allan E., Parkin IP. 2018. White-Light-Activated Antibacterial Surfaces Generated by Synergy between Zinc Oxide Nanoparticles and Crystal Violet. *ACS Omega* 3:3190–3199. DOI: 10.1021/acsomega.7b01473.

Padmavathy N., Samantaray PK., Ghosh LD., Madras G., Bose S. 2017. Selective cleavage of the polyphosphoester in crosslinked copper based nanogels: enhanced antibacterial performance through controlled release of copper. *Nanoscale* 9:12664–12676. DOI: 10.1039/C7NR02446K.

Pang X., Zhitomirsky I. 2008. Electrodeposition of hydroxyapatite–silver–chitosan nanocomposite coatings. *Surface and Coatings Technology* 202:3815–3821. DOI: 10.1016/j.surfcoat.2008.01.022.

Pebdeni AB., Sadri M., Pebdeni SB. 2016. Synthesis of chitosan/PEO/silica nanofiber coating for controlled release of cefepime from implants. *RSC Advances* 6:24418–24429. DOI: 10.1039/C6RA00071A.

Peres RS., Baldissera AF., Armelin E., Alemán C., Ferreira CA. 2014. Marine-friendly antifouling coating based on the use of a fatty acid derivative as a pigment. *Materials Research* 17:720–727. DOI: 10.1590/S1516-14392014005000032.

Pérez-Anes A., Gargouri M., Laure W., Van Den Berghe H., Courcot E., Sobocinski J., Tabary N., Chai F., Blach J-F., Addad A., Woisel P., Douroumis D., Martel B., Blanchemain N., Lyskawa J. 2015. Bioinspired Titanium Drug Eluting Platforms Based on a Poly-β-cyclodextrin–Chitosan Layer-by-Layer Self-Assembly Targeting Infections. *ACS Applied Materials & Interfaces* 7:12882–12893. DOI: 10.1021/acsami.5b02402.

Pérez-Köhler B., Fernández-Gutiérrez M., Pascual G., García-Moreno F., San Román J., Bellón JM. 2016. In vitro assessment of an antibacterial quaternary ammonium-based polymer loaded with chlorhexidine for the coating of polypropylene prosthetic meshes. *Hernia* 20:869–878. DOI: 10.1007/s10029-016-1537-z.

Pfeufer NY., Hofmann-Peiker K., Mühle M., Warnke PH., Weigel MC., Kleine M. 2011. Bioactive Coating of Titanium Surfaces with Recombinant Human &b.beta;-Defensin-2 (rHu&b.beta;D2) May Prevent Bacterial Colonization in Orthopaedic Surgery: *The Journal of Bone and Joint Surgery-American Volume* 93:840–846. DOI: 10.2106/JBJS.I.01738.

Pitarresi G., Palumbo FS., Calascibetta F., Fiorica C., Di Stefano M., Giammona G. 2013. Medicated hydrogels of hyaluronic acid derivatives for use in orthopedic field. *International Journal of Pharmaceutics* 449:84–94. DOI: 10.1016/j.ijpharm.2013.03.059.

Raman N., Marchillo K., Lee M-R., Rodríguez López A de L., Andes DR., Palecek SP., Lynn DM. 2016. Intraluminal Release of an Antifungal β-Peptide Enhances the Antifungal and Anti-Biofilm Activities of Multilayer-Coated Catheters in a Rat Model of Venous Catheter Infection. *ACS Biomaterials Science & Engineering* 2:112–121. DOI: 10.1021/acsbiomaterials.5b00427.

Rubin HN., Neufeld BH., Reynolds MM. 2018. Surface-Anchored Metal–Organic Framework–Cotton Material for Tunable Antibacterial Copper Delivery. *ACS Applied Materials & Interfaces* 10:15189–15199. DOI: 10.1021/acsami.7b19455.

Saif MJ., Zia KM., Rehman F., Ahmad MN., Kiran S., Gulzar T. 2015. An eco-friendly, permanent, and non-leaching antimicrobial coating on cotton fabrics. *The Journal of The Textile Institute* 106:907–911. DOI: 10.1080/00405000.2014.952137.

Saini S., Yücel Falco Ç., Belgacem MN., Bras J. 2016. Surface cationized cellulose nanofibrils for the production of contact active antimicrobial surfaces. *Carbohydrate Polymers* 135:239–247. DOI: 10.1016/j.carbpol.2015.09.002.

Santos-Coquillat A., Gonzalez Tenorio R., Mohedano M., Martinez-Campos E., Arrabal R., Matykina E. 2018. Tailoring of antibacterial and osteogenic properties of Ti6Al4V by plasma electrolytic oxidation. *Applied Surface Science* 454:157–172. DOI: 10.1016/j.apsusc.2018.04.267.

Sathya S., Murthy PS., Das A., Gomathi Sankar G., Venkatnarayanan S., Pandian R., Sathyaseelan VS., Pandiyan V., Doble M., Venugopalan VP. 2016. Marine antifouling property of PMMA nanocomposite films: Results of laboratory and field assessment. *International Biodeterioration & Biodegradation* 114:57–66. DOI: 10.1016/j.ibiod.2016.05.026.

Schiff K., Diehl D., Valkirs A. 2004. Copper emissions from antifouling paint on recreational vessels. *Marine Pollution Bulletin* 48:371–377. DOI: 10.1016/j.marpolbul.2003.08.016.

Sinclair KD., Pham TX., Farnsworth RW., Williams DL., Loc-Carrillo C., Horne LA., Ingebretsen SH., Bloebaum RD. 2012. Development of a broad spectrum polymer-released antimicrobial coating for the prevention of resistant strain bacterial infections. *Journal of Biomedical Materials Research Part A* 100A:2732–2738. DOI: 10.1002/jbm.a.34209.

Sugii MM., Ferreira FA de S., Müller KC., Lima DANL., Groppo FC., Imasato H., Rodrigues-Filho UP., Aguiar FHB. 2017. Physical, chemical and antimicrobial evaluation of a composite material containing quaternary ammonium salt for braces cementation. *Materials Science and Engineering: C* 73:340–346. DOI: 10.1016/j.msec.2016.12.084.

Sun Z., Gu L., Zheng J., Zhang J., Wang L., Xu F., Lin C. 2016. A controlled release strategy of antifouling agent in coating based on intercalated layered double hydroxides. *Materials Letters* 172:105–108. DOI: 10.1016/j.matlet.2016.02.151.

Swanson TE., Cheng X., Friedrich C. 2011. Development of chitosan-vancomycin antimicrobial coatings on titanium implants. *Journal of Biomedical Materials Research Part A* 97A:167–176. DOI: 10.1002/jbm.a.33043.

Taglietti A., Grisoli P., Dacarro G., Gattesco A., Mangano C., Pallavicini P. 2018. Grafted monolayers of the neutral Cu( ii ) complex of a dioxo-2,3,2 ligand: surfaces with decreased antibacterial action. *New Journal of Chemistry* 42:7595–7598. DOI: 10.1039/C7NJ04601D.

Thomas K., Raymond K., Chadwick J., Waldock M. 1999. The effects of short‐term changes in environmental parameters on the release of biocides from antifouling coatings: cuprous oxide and tributyltin. *Applied Organometallic Chemistry* 13:453–460. DOI: 10.1002/(SICI)1099-0739(199906)13:6<453::AID-AOC864>3.0.CO;2-O.

Travan A., Marsich E., Donati I., Benincasa M., Giazzon M., Felisari L., Paoletti S. 2011. Silver–polysaccharide nanocomposite antimicrobial coatings for methacrylic thermosets. *Acta Biomaterialia* 7:337–346. DOI: 10.1016/j.actbio.2010.07.024.

Valkirs AO., Seligman PF., Haslbeck E., Caso JS. 2003. Measurement of copper release rates from antifouling paint under laboratory and in situ conditions: implications for loading estimation to marine water bodies. *Marine Pollution Bulletin* 46:763–779. DOI: 10.1016/S0025-326X(03)00044-4.

Wang B., Liu H., Wang Z., Shi S., Nan K., Xu Q., Ye Z., Chen H. 2017a. A self-defensive antibacterial coating acting through the bacteria-triggered release of a hydrophobic antibiotic from layer-by-layer films. *Journal of Materials Chemistry B* 5:1498–1506. DOI: 10.1039/C6TB02614A.

Wang J., Wu G., Liu X., Sun G., Li D., Wei H. 2017b. A decomposable silica-based antibacterial coating for percutaneous titanium implant. *International Journal of Nanomedicine* Volume 12:371–379. DOI: 10.2147/IJN.S123622.

Wang Y., Zhang D. 2012. Hetero-nanostructured film of titania nanosheets and lysozyme: Fabrication and synergistic antibacterial properties. *Surface and Coatings Technology* 210:71–77. DOI: 10.1016/j.surfcoat.2012.08.066.

Wang Z., Zhang H., Pan G. 2016. Ecotoxicological assessment of flocculant modified soil for lake restoration using an integrated biotic toxicity index. *Water Research* 97:133–141. DOI: 10.1016/j.watres.2015.08.033.

Watermann BT., Daehne B., Sievers S., Dannenberg R., Overbeke JC., Klijnstra JW., Heemken O. 2005. Bioassays and selected chemical analysis of biocide-free antifouling coatings. *Chemosphere* 60:1530–1541. DOI: 10.1016/j.chemosphere.2005.02.066.

Wei X., Yang Z., Tay SL., Gao W. 2014a. Photocatalytic TiO2 nanoparticles enhanced polymer antimicrobial coating. *Applied Surface Science* 290:274–279. DOI: 10.1016/j.apsusc.2013.11.067.

Wei X., Yang Z., Wang Y., Tay SL., Gao W. 2014b. Polymer antimicrobial coatings with embedded fine Cu and Cu salt particles. *Applied Microbiology and Biotechnology* 98:6265–6274. DOI: 10.1007/s00253-014-5670-2.

Wei T., Zhan W., Cao L., Hu C., Qu Y., Yu Q., Chen H. 2016. Multifunctional and Regenerable Antibacterial Surfaces Fabricated by a Universal Strategy. *ACS Applied Materials & Interfaces* 8:30048–30057. DOI: 10.1021/acsami.6b11187.

Wei T., Zhan W., Yu Q., Chen H. 2017. Smart Biointerface with Photoswitched Functions between Bactericidal Activity and Bacteria-Releasing Ability. *ACS Applied Materials & Interfaces* 9:25767–25774. DOI: 10.1021/acsami.7b06483.

Wood NJ., Jenkinson HF., Davis SA., Mann S., O’Sullivan DJ., Barbour ME. 2015. Chlorhexidine hexametaphosphate nanoparticles as a novel antimicrobial coating for dental implants. *Journal of Materials Science: Materials in Medicine* 26. DOI: 10.1007/s10856-015-5532-1.

Xu Q., Liu H., Ye Z., Nan K., Lin S., Chen H., Wang B. 2017. Antimicrobial efficiency of PAA/(PVP/CHI) erodible polysaccharide multilayer through loading and controlled release of antibiotics. *Carbohydrate Polymers* 161:53–62. DOI: 10.1016/j.carbpol.2016.12.054.

Yagci MB., Bolca S., Heuts JPA., Ming W., de With G. 2011a. Self-stratifying antimicrobial polyurethane coatings. *Progress in Organic Coatings* 72:305–314. DOI: 10.1016/j.porgcoat.2011.04.021.

Yagci MB., Bolca S., Heuts JPA., Ming W., de With G. 2011b. Antimicrobial polyurethane coatings based on ionic liquid quaternary ammonium compounds. *Progress in Organic Coatings* 72:343–347. DOI: 10.1016/j.porgcoat.2011.05.006.

Yan S., Song L., Luan S., Xin Z., Du S., Shi H., Yuan S., Yang Y., Yin J. 2017. A hierarchical polymer brush coating with dual-function antibacterial capability. *Colloids and Surfaces B: Biointerfaces* 150:250–260. DOI: 10.1016/j.colsurfb.2016.08.033.

Yang M., Gu L., Yang B., Wang L., Sun Z., Zheng J., Zhang J., Hou J., Lin C. 2017. Antifouling composites with self-adaptive controlled release based on an active compound intercalated into layered double hydroxides. *Applied Surface Science* 426:185–193. DOI: 10.1016/j.apsusc.2017.07.207.

Yang WJ., Pranantyo D., Neoh K-G., Kang E-T., Teo SL-M., Rittschof D. 2012. Layer-by-Layer Click Deposition of Functional Polymer Coatings for Combating Marine Biofouling. *Biomacromolecules* 13:2769–2780. DOI: 10.1021/bm300757e.

Yeasmin R., Zhang H., Zhu J., Kazemian H. 2016. Pre-treatment and conditioning of chabazites followed by functionalization for making suitable additives used in antimicrobial ultra-fine powder coated surfaces. *RSC Advances* 6:88340–88349. DOI: 10.1039/C6RA14295H.

Ytreberg E., Lagerström M., Holmqvist A., Eklund B., Elwing H., Dahlström M., Dahl P., Dahlström M. 2017. A novel XRF method to measure environmental release of copper and zinc from antifouling paints. *Environmental Pollution* 225:490–496. DOI: 10.1016/j.envpol.2017.03.014.

Zaatreh S., Haffner D., Strauß M., Wegner K., Warkentin M., Lurtz C., Zamponi C., Mittelmeier W., Kreikemeyer B., Willumeit-Römer R., Quandt E., Bader R. 2017. Fast corroding, thin magnesium coating displays antibacterial effects and low cytotoxicity. *Biofouling* 33:294–305. DOI: 10.1080/08927014.2017.1303832.

Zhao J., Millians W., Tang S., Wu T., Zhu L., Ming W. 2015. Self-Stratified Antimicrobial Acrylic Coatings via One-Step UV Curing. *ACS Applied Materials & Interfaces* 7:18467–18472. DOI: 10.1021/acsami.5b04633.

Zhou H., Li J., Bao S., Li J., Liu X., Jin P. 2016. Use of ZnO as antireflective, protective, antibacterial, and biocompatible multifunction nanolayer of thermochromic VO2 nanofilm for intelligent windows. *Applied Surface Science* 363:532–542. DOI: 10.1016/j.apsusc.2015.12.045.
